# Standard Vocabularies to Improve Machine Learning Model Transferability With Electronic Health Record Data: Retrospective Cohort Study Using Health Care–Associated Infection

**DOI:** 10.2196/39057

**Published:** 2022-08-30

**Authors:** Amber C Kiser, Karen Eilbeck, Jeffrey P Ferraro, David E Skarda, Matthew H Samore, Brian Bucher

**Affiliations:** 1 Department of Biomedical Informatics School of Medicine University of Utah Salt Lake City, UT United States; 2 Department of Medicine School of Medicine University of Utah Salt Lake City, UT United States; 3 Center for Value-Based Surgery Intermountain Healthcare Salt Lake City, UT United States; 4 Department of Surgery School of Medicine University of Utah Salt Lake City, UT United States; 5 Informatics, Decision-Enhancement and Analytic Sciences Center 2.0 Veterans Affairs Salt Lake City Health Care System Salt Lake City, UT United States

**Keywords:** standard vocabularies, machine learning, electronic health records, model transferability, data heterogeneity, machine learning

## Abstract

**Background:**

With the widespread adoption of electronic healthcare records (EHRs) by US hospitals, there is an opportunity to leverage this data for the development of predictive algorithms to improve clinical care. A key barrier in model development and implementation includes the external validation of model discrimination, which is rare and often results in worse performance. One reason why machine learning models are not externally generalizable is data heterogeneity. A potential solution to address the substantial data heterogeneity between health care systems is to use standard vocabularies to map EHR data elements. The advantage of these vocabularies is a hierarchical relationship between elements, which allows the aggregation of specific clinical features to more general grouped concepts.

**Objective:**

This study aimed to evaluate grouping EHR data using standard vocabularies to improve the transferability of machine learning models for the detection of postoperative health care–associated infections across institutions with different EHR systems.

**Methods:**

Patients who underwent surgery from the University of Utah Health and Intermountain Healthcare from July 2014 to August 2017 with complete follow-up data were included. The primary outcome was a health care–associated infection within 30 days of the procedure. EHR data from 0-30 days after the operation were mapped to standard vocabularies and grouped using the hierarchical relationships of the vocabularies. Model performance was measured using the area under the receiver operating characteristic curve (AUC) and *F*_1_-score in internal and external validations. To evaluate model transferability, a difference-in-difference metric was defined as the difference in performance drop between internal and external validations for the baseline and grouped models.

**Results:**

A total of 5775 patients from the University of Utah and 15,434 patients from Intermountain Healthcare were included. The prevalence of selected outcomes was from 4.9% (761/15,434) to 5% (291/5775) for surgical site infections, from 0.8% (44/5775) to 1.1% (171/15,434) for pneumonia, from 2.6% (400/15,434) to 3% (175/5775) for sepsis, and from 0.8% (125/15,434) to 0.9% (50/5775) for urinary tract infections. In all outcomes, the grouping of data using standard vocabularies resulted in a reduced drop in AUC and *F*_1_-score in external validation compared to baseline features (all *P*<.001, except urinary tract infection AUC: *P*=.002). The difference-in-difference metrics ranged from 0.005 to 0.248 for AUC and from 0.075 to 0.216 for *F*_1_-score.

**Conclusions:**

We demonstrated that grouping machine learning model features based on standard vocabularies improved model transferability between data sets across 2 institutions. Improving model transferability using standard vocabularies has the potential to improve the generalization of clinical prediction models across the health care system.

## Introduction

The widespread adoption of electronic healthcare records (EHRs) by US hospitals has created an opportunity to leverage this data for the development of predictive algorithms to improve clinical care [1]. Various machine learning (ML) models have been developed to predict a variety of outcomes, including pneumonia, sepsis, and surgical site infection [2-5]. However, relatively few of these models have been implemented into clinical practice [6]. A key barrier in model development includes the validation of model discrimination across data sets [7]. Typically, validation occurs using a blind subset of data from the training data set, termed internal validation. External validation using data from a different institution is rare and often results in worse performance [8,9].

There are many reasons why ML models are not externally generalizable, including inadequate training data, overfitting of the model, and data heterogeneity [10]. With 684 different EHR vendors in the United States, the lack of interoperability between institutions, even among those with the same EHR system, substantially inhibits ML model generalizability [11]. Various methods have been proposed to improve the generalizability of ML models, including transfer learning, deep learning, and common data models (CDMs) [9,12-16]. However, data heterogeneity is an underappreciated key determinant of model transferability [17]. Data heterogeneity deriving from variation in laboratory practices, hospital medication formularies, and administrative coding practices between health care systems can impact model performance during external validation, resulting in a decreased transferability of models across institutions [18].

**Figure 1:**
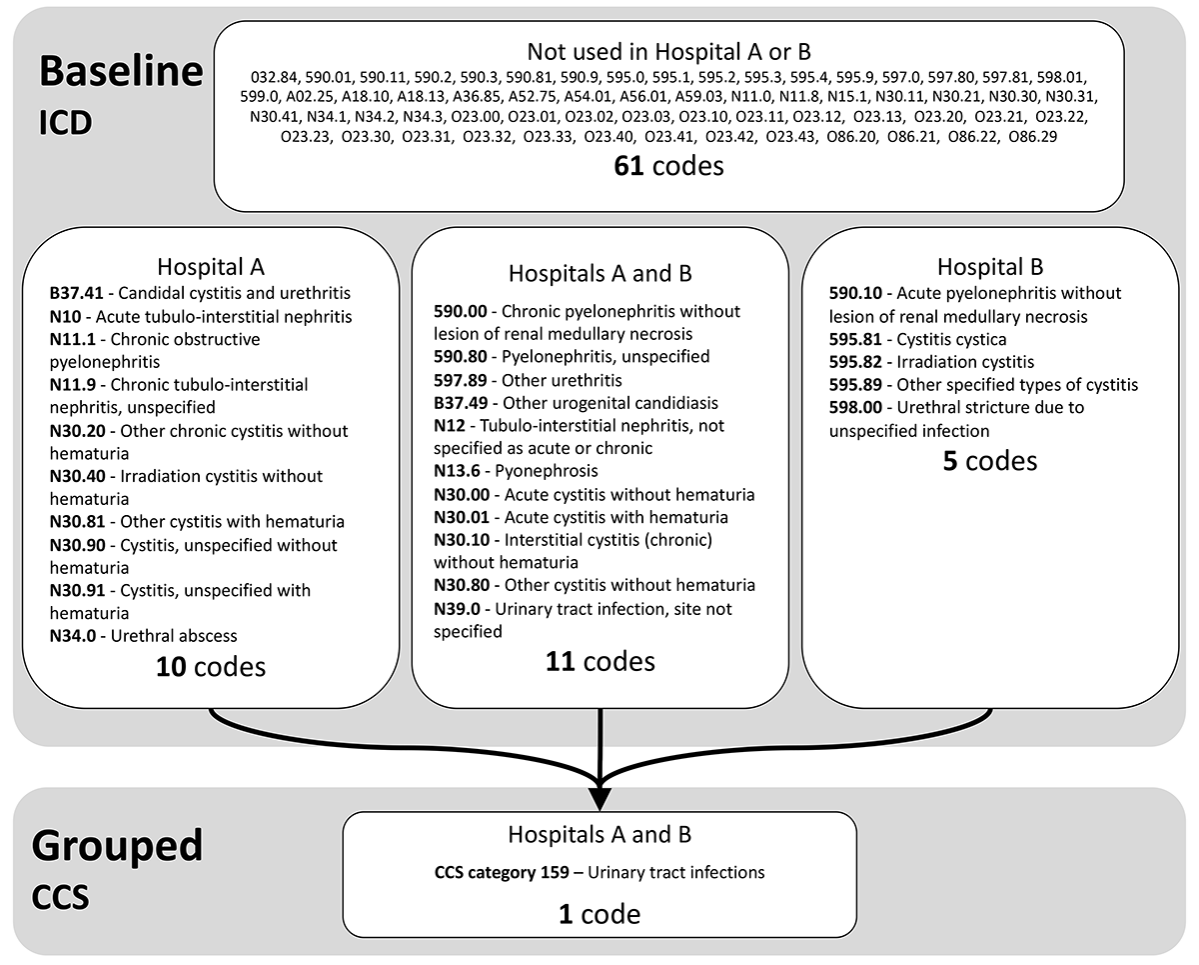
Example of the aggregation of baseline features to grouped concepts. Multiple ICD diagnosis codes describing “urinary tract infections,” including 10 used only in Hospital A, 5 used only in Hospital B, 11 used at both Hospital A and B, and 61 not used in either hospital, can be aggregated to 1 single CCS code. CCS: Clinical Classification Software; ICD: International Classification of Diseases.

A solution to address the substantial data heterogeneity between health care systems is to use standard vocabularies to map EHR data elements. These vocabularies, such as the Clinical Classification Software (CCS) for International Classification of Diseases (ICD) Diagnosis Codes, Logical Observation Identifiers Names and Codes (LOINC) for health care observations, and Medi-Span for medications, can be used to support data harmonization between data sets [19-23]. The advantage of these vocabularies is a hierarchical relationship between elements, which allows the aggregation of specific clinical features to more general grouped concepts. For example, Figure 1 demonstrates how multiple ICD diagnosis codes describing “urinary tract infections” can be aggregated to 1 single CCS code. Due to variation in coding practices among health care facilities, the aggregation of concepts may improve ML model transferability during external validation.

This study’s objective was to evaluate whether aggregating EHR data elements using standard vocabularies would improve ML model transferability to an external data set. Although other works have used this method of grouping EHR data elements when developing ML models, none to our knowledge have assessed the impact of grouping on model transferability to an external data set [17]. To evaluate this objective, we classified postoperative health care–associated infections (HAIs) using EHR data from 2 independent health care systems.

HAIs pose a substantial patient safety concern, raise costs, and increase the risk of death after surgical procedures. HAIs occur in 3% to 27% of surgical patients [24,25]. Developing even 1 major postoperative complication increases a patient’s risk of postoperative mortality and readmission [26,27]. To address the challenges of HAIs, hospitals rely on surveillance programs to monitor HAI rates and develop targeted interventions to address postoperative HAIs. Hospitals that participate in quality surveillance programs reduce HAIs over time [28]. However, hospital surveillance programs rely on a manual chart review process, which is a critical barrier to the widespread adoption of surgical quality assurance programs. To overcome these difficulties, automated surveillance programs are needed to decrease the burden of the manual chart review process [29,30]. We hypothesized that ML models for HAI detection using grouped features from EHR data would improve model performance during external validation compared to ML models developed using baseline features.

## Methods

### Setting

We performed a retrospective cohort study using data from 2 independent health care systems: the University of Utah Health (Hospital A) with an Epic EHR and Intermountain Healthcare (Hospital B) with a Cerner EHR.

### Ethics Approval

The institutional review boards at each health care system approved the study (University of Utah Health: 87482; Intermountain Healthcare: 1050851), granting a waiver of informed consent.

### Data Sources, Participants, and Outcomes

Data for the study were obtained from the American College of Surgeons (ACS) National Surgical Quality Improvement Program (NSQIP) at each institution. The ACS NSQIP program is the largest surgical quality assessment program in the United States, found in over 450 hospitals [31]. As part of the program, the surgical clinical reviewers, typically nurses, are trained in NSQIP methodology and definitions [32]. NSQIP surgical clinical reviewers manually review the EHR records for all selected operative episodes to identify perioperative complications, including HAI, occurring within 30 days of the operation. All identified complications are rereviewed by the ACS surgeon champion at the participating hospital to ensure that the complications meet the ACS NSQIP definitions. Disagreements are settled when a consensus is reached, with the ACS surgeon acting as adjudicator. The interrater reliability and data quality of the NSQIP program have been previously documented [32].

For this study, patient operative episodes were included if they underwent manual chart review as part of the ACS NSQIP program at each institution. Operative events were excluded if they had incomplete follow-up data.

The following HAIs were chosen as outcomes due to their prevalence and clinical relevance: surgical site infection (SSI), pneumonia, sepsis, and urinary tract infection (UTI). These outcomes were selected as they are the most common complications occurring after general and thoracic surgical procedures [33]. In addition, these complications are the most common underlying cause for hospital readmission after surgical procedures [27]. Cases were defined according to standard NSQIP definitions and labeled as binary values for classification.

### EHR Data Element Mapping

**Figure 2:**
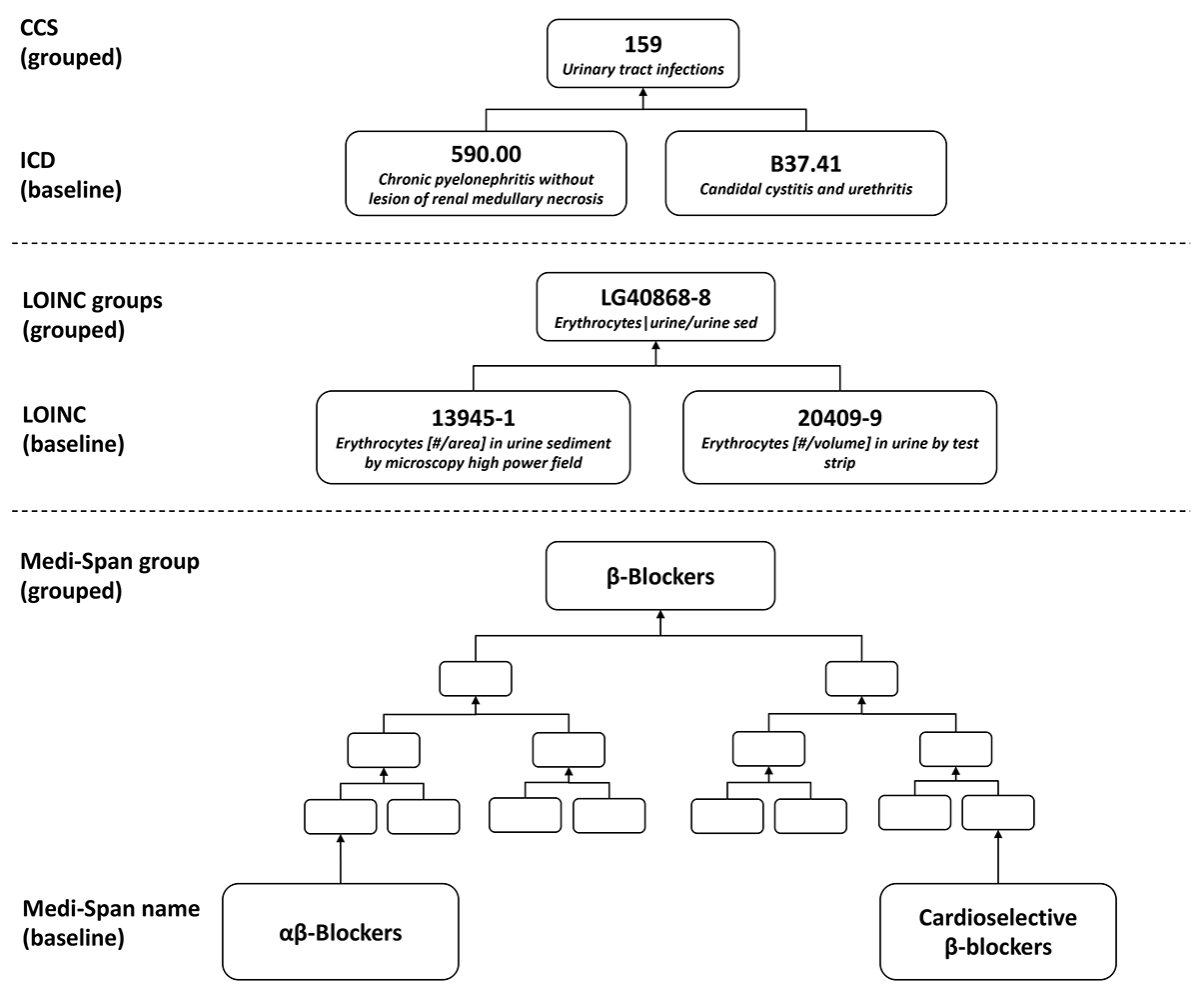
Example of data aggregation. ICD diagnosis codes were manually aggregated into single-level CCS codes. LOINC observations were aggregated into LOINC groups, consisting of a single possible level. Medi-Span consisted of 5 different possible levels of aggregation. Medi-Span drug names were grouped into the highest level of aggregation—Medi-Span drug groups. CCS: Clinical Classification Software; ICD: International Classification of Disease; LOINC: Logical Observation Identifiers Names and Codes.

For selected operative events, we obtained all laboratory test results, medication administration, and ICD 9th and 10th editions diagnosis codes from the EHR between 0-30 days after surgery. Although diagnosis codes are an important indicator of HAI, they often suffer from low sensitivity [34,35]. We chose to include additional clinical features, including laboratory tests and medications, to increase the sensitivity of our models. Each data category was mapped to a standard vocabulary and grouped based on the hierarchical relationships within the standard vocabularies. The Agency for Healthcare Research and Quality provides a mapping from both ICD-9 and ICD-10 codes to CCS codes in the form of a CSV file [19,20]. Diagnosis codes, represented as ICD codes in the EHR, were manually aggregated into single-level CCS codes using the CCS mapping. Laboratory test results were manually mapped to the LOINC terminology and then aggregated into LOINC groups [21,22]. Medications were automatically mapped to the Medi-Span Generic Product Identifier within the EHR [23]. In the Medi-Span hierarchy, we categorized the lowest level as baseline and the highest level as grouped. Figure 2 provides examples of aggregation for each data category. Once mapped, we created 2 discrete data sets. The baseline data set consisted of ICD codes, LOINC tests, and Medi-Span drug names. The grouped data set consisted of aggregated features, including CCS codes, LOINC groups, and Medi-Span drug groups.

### Model Development

**Figure 3:**
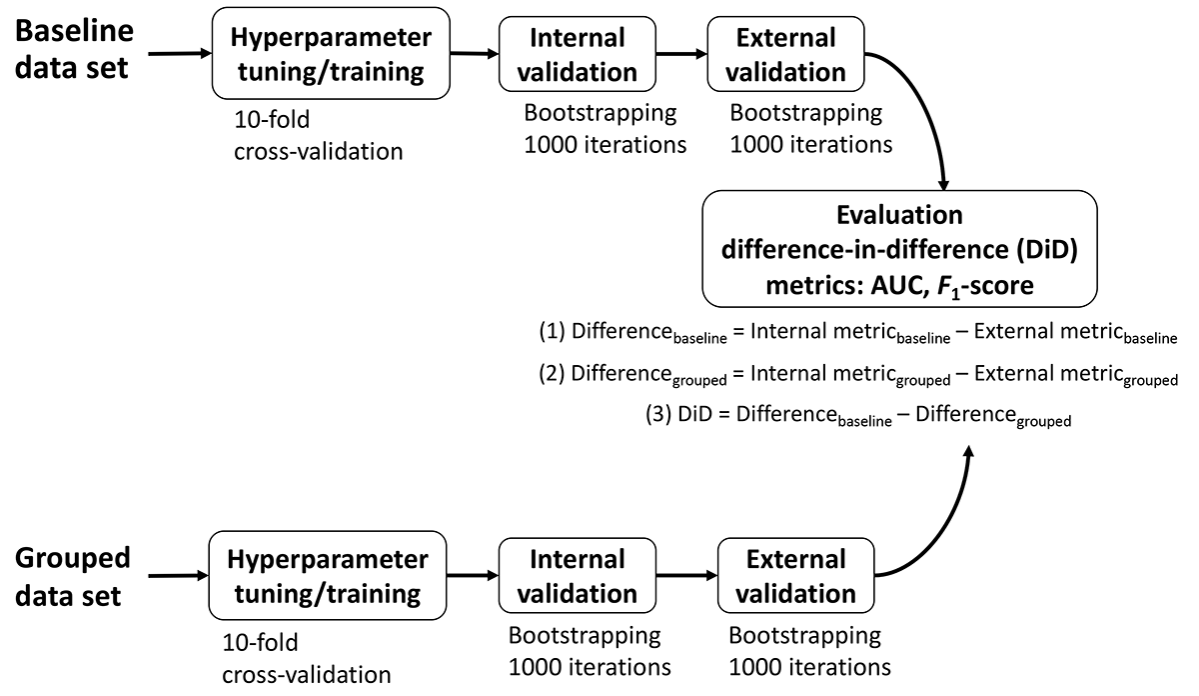
Flow of data through the study with the derivation for the final difference-in-difference (DiD) metric. Final evaluation steps to calculate the DiD included (1) performance difference between the internal and external validations for the baseline model; (2) performance difference between the internal and external validations for the grouped model; and (3) difference in the performance differences between the baseline and grouped models. AUC: area under the receiver operating characteristic curve.

To avoid data leakage and overfitting, we divided the data from Hospital A into hyperparameter tuning/training (70%) and internal validation (30%) data sets before preprocessing or model development. For external validation, we used 100% of the data from Hospital B. Missing data were addressed by imputing 0 for nominal variables and the median value—calculated from the training data—for continuous variables [36]. Data were standardized to have a mean of 0 and SD of 1. Figure 3 briefly describes the flow of the data through model development, validation, and final evaluation.

Separate models were developed for each outcome and data set (baseline or grouped). Each model classified whether an operative event resulted in the relevant HAI within 30 days. Important features were identified based on the ANOVA *F*-score. Data sets with different numbers of *n*-top important features were created. In all, 4 ML algorithms were evaluated: random forest, support vector machine, logistic regression, and XGBoost [37-41]. The number of features and algorithm were included as parameters in model tuning. For each model, tuning was performed using 10-fold cross-validation to improve the internal training. The best model was selected using the area under the receiver operating characteristic curve (AUC) and *F*_1_-score [42,43]. The final training of the models was completed using the whole training data set. To address the class imbalance, random undersampling was used during tuning within each fold of cross-validation and during final training [44]. We did not perform any balancing during validation as we wanted to test in an environment similar to real-life data where we would expect an imbalance. Model development was completed using Python software (version 3.7; Python Software Foundation) and the *scikit-learn* (version 0.22.1), *imblearn* (version 0.6.2), and *xgboost* (version 1.2.1) packages [41,45,46].

### Validation

For each model, we performed internal and external validations. For each outcome, we calculated the difference-in-difference (DiD) defined in Figure 3. DiD is a metric previously used in economics to evaluate the difference in means between 2 groups, generally a control group and an intervention group [47]. We applied it in our study to assess the difference in performance between the baseline and grouped models. A positive DiD indicates that the model developed using grouped features resulted in a reduced drop in performance during external validation compared to the model developed using baseline features.

### Sensitivity Analyses

#### Analysis of Nonshared Codes

A separate granular data set, including baseline features but restricted to those shared by both hospital systems, was created to investigate the magnitude of performance drop in external validation attributable to nonshared codes. Training and validation were conducted as previously described. We calculated the DiD as described in Figure 3.

#### Analysis of Grouping Individual Categories of Data

We investigated the effect of grouping individual data categories, using only SSI, as this outcome was the most prevalent in the data. Training and validation were conducted as previously described. We compared the baseline model with models developed using data sets created with different combinations of baseline and grouped data. The combination data sets were (1) baseline diagnosis codes and laboratory tests with grouped medications, (2) baseline diagnosis codes and medications with grouped laboratory tests, and (3) grouped diagnosis codes with baseline laboratory tests and medications. We calculated the DiD as described in Figure 3.

### Statistical Analysis

We performed a chi-square test of independence to determine any differences in the prevalence of the outcomes and categorical demographic variables between the institutions. For continuous demographic variables, we performed a 2-tailed, 2-sample *t* test to determine any differences between institutions. To measure model performance, bootstrapping for 1000 iterations was used to measure the mean with 95% CIs [48,49]. A 1-tailed, 1-sample *t* test was used to evaluate whether DiD metrics were significantly greater than 0. All statistical tests were completed using the *SciPy* package in Python [50].

## Results

### Cohort and Feature Description

A total of 5775 operative events were retrieved from Hospital A, whereas a total of 15,434 operative events were retrieved from Hospital B. Table 1 describes the study demographics.

**Table 1:** Study demographics for both internal and external data sets.

Characteristic			Hospital A (internal; N=5775)	Hospital B (external; N=15,434)	*P* value
Age at time of surgery (years), mean (SD)			52.6 (16.6)	53.4 (18.1)	.01
Gender, male, n (%)			2765 (47.9)	7576 (49.1)	.12
**Race, n (%)**					
	American Indian or Alaska Native		86 (1.5)	59 (0.4)	<.001
	Asian		81 (1.4)	192 (1.2)	.40
	Black or African American		65 (1.1)	127 (0.8)	.05
	Native Hawaiian or Pacific Islander		34 (0.6)	147 (1)	.05
	White		5275 (91.3)	14,216 (92.1)	.07
	Unknown or not reported		234 (4.1)	693 (4.5)	.18
Ethnicity, Hispanic, n (%)			575 (10)	1384 (9)	.03
**Procedure Current Procedural Terminology code, n (%)**					
	0-29999 (skin/soft tissue)		968 (16.8)	2020 (13.1)	<.001
	30000-39999 (cardiovascular)		594 (10.3)	2222 (14.4)	<.001
	40000-49999 (gastrointestinal)		4172 (72.2)	10,796 (69.9)	.001
	50000-59999 (genitourinary)		27 (0.5)	99 (0.6)	.17
	60000-69999 (nervous system)		14 (0.2)	297 (1.9)	<.001
Inpatient or outpatient status, inpatient, n (%)			2831 (49)	7837 (50.8)	.02
**Comorbidities, n (%)**					
		Diabetes mellitus	822 (14.2)	2144 (13.9)	.54
		Current smoker within 1 year	799 (13.8)	2248 (14.6)	.18
		Dyspnea	498 (8.6)	373 (2.4)	<.001
		Functional heath status	71 (1.2)	376 (2.4)	<.001
		Being ventilator-dependent	20 (0.3)	149 (1)	<.001
		History of severe chronic obstructive pulmonary disease	128 (2.2)	417 (2.7)	.05
		Ascites within 30 days prior to surgery	8 (0.1)	114 (0.7)	<.001
		Congestive heart failure within 30 days prior to surgery	24 (0.4)	123 (0.8)	.004
		Hypertension requiring medication	1940 (33.6)	5455 (35.3)	.02
		Acute renal failure	9 (0.2)	53 (0.3)	.03
		Currently requiring or on dialysis	100 (1.7)	283 (1.8)	.66
		Disseminated cancer	187 (3.2)	246 (1.6)	<.001
		Open wound with or without infection	287 (5)	512 (3.3)	<.001
		Steroid or immunosuppressant use for chronic condition	351 (6.1)	644 (4.2)	<.001
		>10% loss of body weight in the 6 months prior to surgery	145 (2.5)	372 (2.4)	.71
		Bleeding disorder	151 (2.6)	1013 (6.6)	<.001

Table 2 describes the prevalence of HAI outcomes within each institution. There were no significant differences in the prevalence of SSI (*P*=.77), sepsis (*P*=.09), or UTI (*P*=.75). The prevalence of pneumonia was significantly higher (*P*=.03) in Hospital B.

**Table 2:** Prevalence of selected outcomes in each hospital system.

Outcome	Hospital A (N=5775), n (%)	Hospital B (N=15,434), n (%)	*P* value
Surgical site infection	291 (5)	761 (4.9)	.77
Pneumonia	44 (0.8)	171 (1.1)	.03^a^
Sepsis	175 (3)	400 (2.6)	.09
Urinary tract infection	50 (0.9)	125 (0.8)	.75

^a^Pneumonia was significantly more prevalent in Hospital B (*P*<.05).

### Model Development and Validation

DiD metrics are reported in Table 3. Tables S1 and S2 in Multimedia Appendix 1 detail the selected model parameters. Model calibration can be found in Table S3 and Figures S1-S4 in Multimedia Appendix 1. Standards for Reporting Diagnostic Accuracy Studies flow diagrams of patient data through the top-performing models can be seen in Figures S5-S16 in Multimedia Appendix 1.

After external validation, all models produced significantly positive AUC and *F*_1_-score DiDs when comparing the performance of the baseline and grouped models (all *P*<.001, except UTI AUC: *P*=.002). A forest plot in Figure S17 in Multimedia Appendix 1 illustrates the AUC and *F*_1_-score DiDs.

**Table 3:** Difference-in-difference (DiD) metrics for each outcome. Means are based on 1000 bootstrapped iterations with 95% CIs. A positive DiD indicates that the grouped model resulted in a reduced drop in performance compared to the baseline model.

Outcome, metric		Top baseline algorithm	Top grouped algorithm	Baseline internal validation, mean (95% CI)	Baseline external validation, mean (95% CI)	Grouped internal validation, mean (95% CI)	Grouped external validation, mean (95% CI)	DiD, mean (95% CI)	*P* value
**SSI^a^**		SVM^b^	LR^c^						
	AUC^d^			0.906 (0.904-0.908)	0.763 (0.762-0.764)	0.904 (0.903-0.906)	0.833 (0.833-0.834)	0.072 (0.070-0.074)	<.001
	*F_1_*-score			0.501 (0.499-0.503)	0.300 (0.299-0.302)	0.476 (0.474-0.478)	0.376 (0.375-0.376)	0.100 (0.097-0.103)	<.001
**Pneumonia**		LR	SVM						
	AUC			0.953 (0.949-0.957)	0.683 (0.682-0.685)	0.994 (0.994-0.995)	0.973 (0.973-0.974)	0.250 (0.247-0.252)	<.001
	*F_1_*-score			0.504 (0.498-0.509)	0.302 (0.299-0.305)	0.456 (0.452-0.461)	0.467 (0.465-0.468)	0.212 (0.206-0.218)	<.001
**Sepsis**		LR	RF^e^						
	AUC			0.964 (0.963-0.964)	0.890 (0.889-0.891)	0.948 (0.946-0.949)	0.883 (0.883-0.884)	0.008 (0.007-0.010)	<.001
	*F_1_*-score			0.469 (0.467-0.472)	0.050 (0.050-0.050)	0.419 (0.416-0.422)	0.092 (0.092-0.093)	0.091 (0.089-0.093)	<.001
**UTI^f^**		SVM	LR						
	AUC			0.898 (0.895-0.900)	0.886 (0.885-0.887)	0.936 (0.934-0.939)	0.929 (0.928-0.930)	0.006 (0.002-0.009)	.002
	*F_1_*-score			0.153 (0.148-0.158)	0.063 (0.061-0.064)	0.244 (0.241-0.246)	0.225 (0.224-0.226)	0.073 (0.068-0.077)	<.001

^a^SSI: surgical site infection.

^b^SVM: support vector machine.

^c^LR: logistic regression.

^d^AUC: area under the receiver operating characteristic curve.

^e^RF: random forest.

^f^UTI: urinary tract infection.

### Sensitivity Analyses

#### Effect of Nonshared Codes

Table 4 describes the EHR data elements shared between hospitals. We found that 44.8% (4284/9559) of baseline features present in the training set were not present in the external set, whereas all grouped features present in the training set were present in the external set.

After external validation, all models, except UTI (*P*=.002), produced significantly positive AUC DiDs (all *P*<.001) when comparing the performance of the baseline and granular models. All outcomes produced significantly positive *F*_1_-score DiDs (all *P*<.001) when comparing the performance of the baseline and granular models.

The magnitude of the AUC and *F*_1_-score DiDs calculated from the comparison of the baseline and grouped models were greater than those calculated from the comparison of the baseline and granular models in all outcomes, except the AUC DiD for sepsis, as represented in Table 5. Full internal and external validation results can be found in Table S4 in Multimedia Appendix 1.

**Table 4:** Number of features in each category (diagnosis, medication, and laboratory) for Hospital A, Hospital B, and those shared between them.

Features		Training Set (Hospital A), n	External Set (Hospital B), n	Shared, n
**Baseline**				
	Total	9559	7926	5275
	ICD^a^ diagnosis codes	7708	6859	4392
	Medi-Span drug names	1311	531	531
	LOINC^b^ codes	540	536	352
**Grouped**				
	Total	805	817	805
	CCS^c^ diagnosis codes	287	287	287
	Medi-Span drug groups	94	94	94
	LOINC groups	424	436	424

^a^ICD: International Classification of Diseases.

^b^LOINC: Logical Observation Identifiers Names and Codes.

^c^CCS: Clinical Classification Software.

**Table 5:** Difference-in-difference (DiD) metrics for the comparison between baseline and granular models and the comparison between baseline and grouped models. A positive DiD indicates the comparison model resulted in a reduced drop in performance compared to the baseline model.

Metric, outcome		Granular comparison, DiD (95% CI)	Grouped comparison, DiD (95% CI)
**AUC^a^**			
	SSI^b^	0.035 (0.033-0.037)	0.072 (0.070-0.074)
	Pneumonia	0.226 (0.223-0.229)	0.250 (0.247-0.252)
	Sepsis	0.015 (0.013-0.017)	0.008 (0.007-0.010)
	UTI^c^	–0.049 (–0.052 to –0.045)	0.006 (0.002-0.009)
***F_1_*-score**			
	SSI	0.017 (0.014-0.020)	0.100 (0.097-0.103)
	Pneumonia	0.186 (0.179-0.193)	0.212 (0.206-0.218)
	Sepsis	0.026 (0.023-0.028)	0.091 (0.089-0.093)
	UTI	0.039 (0.035-0.043)	0.073 (0.068-0.077)

^a^AUC: area under the receiver operating characteristic curve.

^b^SSI: surgical site infection.

^c^UTI: urinary tract infection.

#### Effect of Grouping Individual Categories of Data

In the second sensitivity analysis, all AUC and *F*_1_-score DiDs were significantly positive (all *P*<.001) when comparing the performance of the baseline and combination models, as displayed in Table 6. The combination model with grouped medications, Combination 1, resulted in the greatest AUC DiD. The combination model with grouped diagnosis codes, Combination 3, resulted in the greatest *F*_1_-score DiD.

**Table 6:** Comparison of models developed from baseline data with models developed from the combination of baseline and grouped data. The difference-in-difference (DiD) reflects the AUC and *F*_1_-score for surgical site infection. A positive DiD indicates the combination model resulted in a smaller drop in performance than the baseline model.

Combination	Medications	Laboratory tests	Diagnosis codes	AUC^a^, DiD (95% CI)	*F*_1_-score, DiD (95% CI)	*P* value
Combination 1	Grouped	Baseline	Baseline	0.054 (0.052-0.057)	0.072 (0.069-0.074)	<.001
Combination 2	Baseline	Grouped	Baseline	0.012 (0.010-0.014)	0.046 (0.043-0.049)	<.001
Combination 3	Baseline	Baseline	Grouped	0.049 (0.047-0.051)	0.134 (0.131-0.137)	<.001

^a^AUC: area under the receiver operating characteristic curve.

## Discussion

We investigated the effect that grouping EHR data using standard vocabularies has on ML model transferability during external validation. There are several novel and significant findings of our work. First, ML models for HAI detection with grouped features based on standard vocabularies resulted in a reduced drop in performance when validated on an external data set compared to baseline features. Second, there was significant heterogeneity of EHR data elements between health care systems, as 45% of data elements present in the training set were not present in the external set. Third, ML models developed from grouped data sets resulted in greater performance gains after external validation compared to data sets restricted to shared codes alone. Lastly, we found that grouping diagnosis codes and medications was important to model transferability when compared to laboratory tests.

We demonstrated that grouping features using standard vocabularies improved model transferability during external validation. We found on average a 51% decrease and 65% decrease in the performance drop of AUC and *F*_1_-score, respectively, during external validation when using grouped data compared to baseline data. This improvement in transferability can be attributed to better syntactic and semantic interoperability. Using grouped features allows the model to overcome the challenges of data heterogeneity, such as differences in coding practice and hospital formularies, that arise when using granular codes. A single feature from the grouped model can represent several distinct features from the baseline model (Figure 1). Hence, this method can generalize to an unknown data set as no knowledge of the future data set is required when selecting features or training the model. Although the practice of grouping features is common, our study is novel in that to our knowledge, previous studies have not evaluated model transferability in an external data set when grouping features based on standard vocabularies.

The data heterogeneity seen in our data highlights the difficulty when creating generalizable ML models. Shared codes accounted for 57% (4392/7708) of the ICD diagnosis codes used in Hospital A and 64% (4392/6859) of the ICD diagnosis codes used in Hospital B. To our knowledge, none have compared ICD code usage between hospitals. For several common conditions, there are numerous ICD diagnosis codes available. For example, diabetes mellitus type II has 56 ICD-9 and ICD-10 codes available [51,52]. Variation in coding practices between health care systems can result in several individual codes not being present in a given data set. Differences in laboratory practices or hospital medication formularies may also contribute to EHR data heterogeneity. Extensive feature engineering is typically performed to overcome this challenge before model development [53]. Feature engineering, while creating highly relevant features for the given use case, represents a substantial barrier to model generalizability. Our study demonstrated that grouping features can overcome challenges created by data heterogeneity.

In the first sensitivity analysis, we found that although models developed with granular data sets restricted to shared codes resulted in a reduced drop in performance when compared to a baseline model, models developed from grouped data sets resulted in an even smaller drop in performance. The models developed using grouped data sets resulted in an additional 41% decrease and 70% decrease in performance drop of AUC and *F*_1_-score, respectively, during external validation on average. These results provide further evidence that grouping features using standard vocabularies produces greater benefits than just restricting features to those shared by other hospital systems.

In the second sensitivity analysis, we found that the most important factors when improving transferability included grouping both diagnosis codes and medications. This result could be explained by the amount of information lost due to variation in coding practices and prescription preferences when using baseline data. Rasmy et al [54] compared models using different representations of diagnosis codes in the EHR. The study found that models developed with data mapped to the Unified Medical Language System (UMLS) produced the highest AUC, whereas models developed with data mapped to CCS codes produced the lowest AUC. However, this previous study did not have an external data set to compare performance.

Other studies have used various methods to improve model transferability, including transfer learning, deep learning, and anchor learning [9,12-16]. Curth et al [12] found that using transfer learning significantly increased model performance, where the AUC increased as much as from 4.7% to 7.0% depending on the use case. Although transfer learning has been shown to be successful, it requires models to be trained with data from the internal and external sites. Rasmy et al [15] found an average drop of 3.6% in AUC when evaluating the generalizability of a recurrent neural network. In our study, we found the average drop in AUC to be 13% in models developed using baseline data but only 4% in models developed using grouped data. Kashyap et al [13] found performance drops in both recall and precision when validating the model at an external site after using anchor learning. Our study evaluated a method to achieve comparable model transferability without requiring any knowledge of the external site or a deep learning model.

Mapping data to CDMs can facilitate the sharing of data and models across institutions as seen in several recent studies [13,55]. Recent work, such as that from Tian et al [9], has built frameworks for model sharing and generalizability that use CDMs in their pipeline [17]. The use of a CDM involves mapping data to standard vocabularies as we did in our study, which addresses the problem of syntax by standardizing the vocabulary. In our study, we further address the problem of semantics, where different hospitals may use the same vocabulary, but coding practices may result in different codes representing the same condition.

We acknowledge several limitations to this study. Our use case consisted of HAI detection in patients who underwent surgery. The benefit of grouping feature sets for ML development may not be consistent across other use cases. We only used EHR data elements for which there are standard vocabularies available, excluding features such as microbiology reports or clinical text. It is likely that including these additional features would improve ML model performance at the expense of requiring an extensive amount of feature engineering. We used Medi-Span, a proprietary vocabulary, as both hospital EHRs mapped medications to this system. Other vocabularies, such as RxNorm, could be used. There are several different terminologies that can be used to group diagnosis codes in addition to CCS, including UMLS, as was studied by Rasmy et al [54]. Their work indicates that using UMLS to group diagnosis codes could produce an even smaller drop in performance than we found with CCS. This method would be a valuable investigation for future studies that could lead to even greater results. The terminologies and levels chosen for our study could be modified for different use cases.

This study has substantial implications for the application of ML models to clinical practice. Significant improvements in patient care can be achieved with ML models as demonstrated in previous studies [13,14,56,57]. However, external validation remains one of the most serious barriers to the widespread use of ML models in clinical practice [6,58]. We found that 2 independent hospitals only shared 55% of baseline EHR data elements, highlighting the difficulty when creating generalizable ML models. Current practices to overcome the data heterogeneity between data sets involve extensive feature engineering, which is burdensome during model deployment at a new health care system where EHR data elements are not mapped to a CDM [59]. We demonstrated the novel finding that grouping features with standard vocabularies can overcome the challenge of data heterogeneity and improve ML model performance in external data sets. The method of grouping features based on standard vocabularies will improve the transferability of models, allowing for more widespread use of these ML models between health care systems.

## Appendix

Multimedia Appendix 1 Supplemental tables and figures.

## Abbreviations

ACS: American College of Surgeons
AUC: area under the receiver operating characteristic curve
CCS: Clinical Classification Software
CDM: common data model
DiD: difference-in-difference
EHR: electronic healthcare records
HAI: health care–associated infection
ICD: International Classification of Diseases
LOINC: Logical Observation Identifiers Names and Codes
ML: machine learning
NSQIP: National Surgical Quality Improvement Program
SSI: surgical site infection
UMLS: Unified Medical Language System4^
UTI: urinary tract infection

## Data Availability

The code for this article can be found in the public GitHub repository amberkiser/MachineLearningTransferability. The data underlying this article cannot be shared publicly as it contains protected health information.
