# Supplementary material for: Standard Vocabularies to Improve Machine Learning Model Transferability With Electronic Health Record Data: Retrospective Cohort Study Using Health Care–Associated Infection
Source: JMIR Med Inform. 2022 Aug 30;10(8):e39057. doi: 10.2196/39057 (PMC9472055; doi:10.2196/39057)
Supplement: Multimedia Appendix 1 [file medinform_v10i8e39057_app1.docx]

## MULTIMEDIA APPENDIX 1

### Tables

Table S1. Description of top performing models, including the algorithm, number of features, and hyperparameter settings. The range for each hyperparameter used for tuning is represented in brackets. Hyperparameters with no brackets were not tuned. Support Vector Machine (SVM); Logistic Regression (LR); Random Forest (RF).

| **Outcome** | **Dataset** | **Algorithm** | **Number of Features** | **Hyperparameter Settings [Hyperparameter Tuning Range]** |
| --- | --- | --- | --- | --- |
| SSI | Baseline | SVM | 10 | probability: True  gamma: scale  kernel: linear [linear, rbf],  C: 0.25 [0.01, 0.1, 0.25, 0.50, 1, 2] |
|  | Granular | LR | 10 | max_iter: 50000  solver: liblinear [saga, liblinear],  penalty: l1 [l1, l2],  C: 0.25 [0.01, 0.1, 0.25, 0.50, 1, 2] |
|  | Grouped | LR | 10 | max_iter: 50000  solver: saga [saga, liblinear],  penalty: l1 [l1, l2],  C: 0.25 [0.01, 0.1, 0.25, 0.50, 1, 2] |
| Pneumonia | Baseline | LR | 5 | max_iter: 50000  solver: liblinear [saga, liblinear],  penalty: l2 [l1, l2],  C: 0.1 [0.01, 0.1, 0.25, 0.50, 1, 2] |
|  | Granular | LR | 25 | max_iter: 50000  solver: liblinear [saga, liblinear],  penalty: l2 [l1, l2],  C: 0.5 [0.01, 0.1, 0.25, 0.50, 1, 2] |
|  | Grouped | SVM | 10 | probability: True  gamma: scale  kernel: linear [linear, rbf],  C: 2 [0.01, 0.1, 0.25, 0.50, 1, 2] |
| Sepsis | Baseline | LR | 1000 | max_iter: 50000  solver: saga [saga, liblinear],  penalty: l2 [l1, l2],  C: 0.01 [0.01, 0.1, 0.25, 0.50, 1, 2] |
|  | Granular | LR | 500 | max_iter: 50000  solver: liblinear [saga, liblinear],  penalty: l2 [l1, l2],  C: 0.01 [0.01, 0.1, 0.25, 0.50, 1, 2] |
|  | Grouped | RF | 10 | n_estimators: 200 [10, 50, 100, 200],  max_depth: 2 [None, 2, 10, 20],  criterion: entropy [gini, entropy] |
| UTI | Baseline | SVM | 100 | probability: True  gamma: scale  kernel: rbf [linear, rbf],  C: 0.1 [0.01, 0.1, 0.25, 0.50, 1, 2] |
|  | Granular | RF | 50 | n_estimators: 200 [10, 50, 100, 200],  max_depth: 20 [None, 2, 10, 20],  criterion: gini [gini, entropy] |
|  | Grouped | LR | 10 | max_iter: 50000  solver: saga [saga, liblinear],  penalty: l1 [l1, l2],  C: 0.25 [0.01, 0.1, 0.25, 0.50, 1, 2] |

Table S2. Description of features (up to 25) for each outcome and dataset. The description for nominal features includes a list of paired value and counts. For diagnosis codes, the values represent the number of times a diagnosis code appears in the EHR for the 30 day period. For medications, the values represent the number of times a medication was administered in the 30 day period. The description for continuous features provides a list of summary descriptors, including range (min, max), mean, and standard deviation.

| **Outcome** | **Dataset** | **Feature** | **Type** | **Category / Terminology** | **Missing Rate** | **Description**  Nominal features: list of [value: count]  Continuous features: [range (min,max), mean, standard deviation] |
| --- | --- | --- | --- | --- | --- | --- |
| SSI | Baseline | ICD T814XXA - Infection following a procedure, initial encounter | nominal | Diagnosis / ICD | 0% | “0”: 5639, “2”: 122, “4”: 12, “6”: 2 |
|  |  | ICD K651 - Peritoneal abscess | nominal | Diagnosis / ICD | 0% | “0”: 5674, “1”: 89, “2”: 10, “3”: 1,  “4”: 1 |
|  |  | Radiographic Contrast Media Iodinated | nominal | Medications /  Medi-Span | 0% | “0”: 4364, “1”: 1098, “2”: 214,  “3”: 63, “4”: 22, “5”: 6, “6”: 3, “7”: 5 |
|  |  | LAB 26515-7 max - Platelet count, Blood | continuous | Laboratory Test Results / LOINC | 44.2% | Range: (46, 1219), Mean: 307.3,  SD: 152.1 |
|  |  | Low Molecular Weight Heparins | nominal | Medications /  Medi-Span | 0% | “0”: 3761, “1”: 258, “2”: 565, “3”: 351, “4”: 223, “5”: 156, “6”: 116, “7”: 78, “8”: 59, “9”: 27, “10”: 33, “11”: 29, “12”: 14, “13”: 21, “14”: 17, “15”: 9, “16”: 6, “17”: 8, “18”: 6, “19”: 10, “20”: 2, “21”: 6, “22”: 4, “23”: 5,  “24”: 3, “25”: 4, “26”: 2, “28”: 1,  “29”: 1 |
|  |  | ICD 99859 - Other postoperative infection | nominal | Diagnosis / ICD | 0% | “0”: 5682, “3”: 83, “6”: 8, “9”: 1,  “10”: 1 |
|  |  | Opioid Agonists | nominal | Medications /  Medi-Span | 0% | “0”: 887, “1”: 2108, “2”: 854, “3”: 655, “4”: 347, “5”: 260, “6”: 150, “7”: 111, “8”: 77, “9”: 57, “10”: 44, “11”: 38, “12”: 31, “13”: 29, “14”: 15, “15”: 16, “16”: 12, “17”: 8, “18”: 10, “19”: 5, “20”: 12, “21”: 5, “22”: 6, “23”: 6,  “24”: 2, “25”: 5, “26”: 4, “27”: 3,  “28”: 4, “29”: 4, “30”: 3, “31”: 7 |
|  |  | Analgesics Other | nominal | Medications /  Medi-Span | 0% | “0”: 2310, “1”: 1124, “2”: 663,  “3”: 563, “4”: 301, “5”: 210, “6”: 143, “7”: 91, “8”: 73, “9”: 43, “10”: 42, “11”: 30, “12”: 38, “13”: 22, “14”: 19, “15”: 18, “16”: 18, “17”: 9, “18”: 8, “19”: 6, “20”: 2, “21”: 7, “22”: 2,  “23”: 5, “24”: 4, “25”: 3, “26”: 3,  “27”: 3, “29”: 1, “30”: 8, “31”: 6 |
|  |  | ICD 56722 - Peritoneal abscess | nominal | Diagnosis / ICD | 0% | “0”: 5728, “1”: 40, “2”: 6, “4”: 1 |
|  |  | ICD K9189 - Other postprocedural complications and disorders of digestive system | nominal | Diagnosis / ICD | 0% | “0”: 5570, “1”: 189, “2”: 14, “3”: 2 |
|  | Granular | ICD T814XXA - Infection following a procedure, initial encounter | nominal | Diagnosis / ICD | 0% | “0”: 5,639, “2”: 122, “4”: 12, “6”: 2 |
|  |  | ICD K651 - Peritoneal abscess | nominal | Diagnosis / ICD | 0% | “0”: 5,674, “1”: 89, “2”: 10, “3”: 1, “4”: 1 |
|  |  | Low Molecular Weight Heparins | nominal | Medications /  Medi-Span | 0% | “0”: 3761, “1”: 258, “2”: 565, “3”: 351, “4”: 223, “5”: 156, “6”: 116, “7”: 78, “8”: 59, “9”: 27, “10”: 33, “11”: 29, “12”: 14, “13”: 21, “14”: 17, “15”: 9, “16”: 6, “17”: 8, “18”: 6, “19”: 10, “20”: 2, “21”: 6, “22”: 4, “23”: 5,  “24”: 3, “25”: 4, “26”: 2, “28”: 1,  “29”: 1 |
|  |  | ICD 99859 - Other postoperative infection | nominal | Diagnosis / ICD | 0% | “0”: 5682, “3”: 83, “6”: 8, “9”: 1,  “10”: 1 |
|  |  | Opioid Agonists | nominal | Medications /  Medi-Span | 0% | “0”: 887, “1”: 2108, “2”: 854, “3”: 655, “4”: 347, “5”: 260, “6”: 150, “7”: 111, “8”: 77, “9”: 57, “10”: 44, “11”: 38, “12”: 31, “13”: 29, “14”: 15, “15”: 16, “16”: 12, “17”: 8, “18”: 10, “19”: 5, “20”: 12, “21”: 5, “22”: 6, “23”: 6,  “24”: 2, “25”: 5, “26”: 4, “27”: 3,  “28”: 4, “29”: 4, “30”: 3, “31”: 7 |
|  |  | Analgesics Other | nominal | Medications /  Medi-Span | 0% | “0”: 2310, “1”: 1124, “2”: 663,  “3”: 563, “4”: 301, “5”: 210, “6”: 143, “7”: 91, “8”: 73, “9”: 43, “10”: 42, “11”: 30, “12”: 38, “13”: 22, “14”: 19, “15”: 18, “16”: 18, “17”: 9, “18”: 8, “19”: 6, “20”: 2, “21”: 7, “22”: 2,  “23”: 5, “24”: 4, “25”: 3, “26”: 3,  “27”: 3, “29”: 1, “30”: 8, “31”: 6 |
|  |  | ICD 56722 - Peritoneal abscess | nominal | Diagnosis / ICD | 0% | “0”: 5728, “1”: 40, “2”: 6, “4”: 1 |
|  |  | ICD K9189 - Other postprocedural complications and disorders of digestive system | nominal | Diagnosis / ICD | 0% | “0”: 5570, “1”: 189, “2”: 14, “3”: 2 |
|  |  | 5HT3 Receptor Antagonists | nominal | Medications /  Medi-Span | 0% | “0”: 314, “1”: 3570, “2”: 857, “3”: 398, “4”: 205, “5”: 145, “6”: 85, “7”: 49, “8”: 29, “9”: 24, “10”: 17, “11”: 24, “12”: 13, “13”: 11, “14”: 7, “15”: 3, “16”: 6, “17”: 8, “18”: 2, “19”: 3,  “21”: 4, “23”: 1 |
|  |  | Irrigation Solutions | nominal | Medications /  Medi-Span | 0% | “0”: 4199, “1”: 1419, “2”: 124, “3”: 21, “4”: 7, “5”: 3, “7”: 1, “8”: 1 |
|  | Grouped | Postoperative Infection | nominal | Diagnosis / CCS | 0% | “0”: 5536, “1”: 209, “2”: 25, “3”: 3, “4”: 1, “7”: 1 |
|  |  | Peritonitis and intestinal abscess | nominal | Diagnosis / CCS | 0% | “0”: 5488, “1”: 262, “2”: 23, “3”: 1, “5”: 1 |
|  |  | Complications of surgical procedures or medical care | nominal | Diagnosis / CCS | 0% | “0”: 4672, “1”: 903, “2”: 156, “3”: 36, “4”: 6, “5”: 1, “7”: 1 |
|  |  | Diagnostic Products | nominal | Medications /  Medi-Span | 0% | “0”: 4120, “1”: 1273, “2”: 247, “3”: 83, “4”: 32, “5”: 8, “6”: 5, “7”: 4, “8”: 3 |
|  |  | LAB LG32892-8 max - Platelets [#/volume] in Blood | continuous | Laboratory Test Results / LOINC | 44.2% | Range: (46, 1219), Mean: 307.3,  SD: 152.1 |
|  |  | Anticoagulants | nominal | Medications /  Medi-Span | 0% | “0”: 2777, “1”: 422, “2”: 492, “3”: 614, “4”: 297, “5”: 243, “6”: 199, “7”: 158, “8”: 105, “9”: 75, “10”: 66, “11”: 43, “12”: 40, “13”: 31, “14”: 35, “15”: 24, “16”: 18, “17”: 17, “18”: 16, “19”: 16, “20”: 14, “21”: 15, “22”: 2, “23”: 8,  “24”: 8, “25”: 9, “26”: 4, “27”: 7,  “28”: 8, “29”: 7, “30”: 4, “31”: 1 |
|  |  | Analgesics Opioid | nominal | Medications /  Medi-Span | 0% | “0”: 568, “1”: 2045, “2”: 937, “3”: 742, “4”: 429, “5”: 302, “6”: 182, “7”: 125, “8”: 84, “9”: 67, “10”: 46, “11”: 40, “12”: 39, “13”: 31, “14”: 18, “15”: 18, “16”: 13, “17”: 8, “18”: 11, “19”: 5, “20”: 13, “21”: 6, “22”: 4, “23”: 7,  “24”: 2, “25”: 7, “26”: 5, “27”: 3,  “28”: 4, “29”: 4, “30”: 3, “31”: 7 |
|  |  | Ulcer Drugs | nominal | Medications /  Medi-Span | 0% | “0”: 2319, “1”: 1697, “2”: 310,  “3”: 430, “4”: 234, “5”: 146, “6”: 115, “7”: 106, “8”: 71, “9”: 66, “10”: 39, “11”: 29, “12”: 36, “13”: 23, “14”: 17, “15”: 16, “16”: 12, “17”: 12, “18”: 10, “19”: 17, “20”: 13, “21”: 5, “22”: 9, “23”: 1, “24”: 5, “25”: 6, “26”: 6,  “27”: 4, “28”: 5, “29”: 7, “30”: 6,  “31”: 3 |
|  |  | Analgesics  Non-Narcotic | nominal | Medications /  Medi-Span | 0% | “0”: 2190, “1”: 1083, “2”: 690,  “3”: 585, “4”: 306, “5”: 220, “6”: 160, “7”: 116, “8”: 80, “9”: 54, “10”: 39, “11”: 30, “12”: 39, “13”: 23, “14”: 25, “15”: 23, “16”: 18, “17”: 9, “18”: 11, “19”: 6, “20”: 8, “21”: 7, “22”: 4,  “23”: 6, “24”: 8, “25”: 3, “26”: 5,  “27”: 6, “28”: 2, “29”: 3, “30”: 9,  “31”: 7 |
|  |  | Antiemetics | nominal | Medications /  Medi-Span | 0% | “0”: 310, “1”: 3567, “2”: 860, “3”: 399, “4”: 206, “5”: 142, “6”: 88, “7”: 46, “8”: 29, “9”: 26, “10”: 19, “11”: 23, “12”: 13, “13”: 13, “14”: 7, “15”: 3, “16”: 6, “17”: 8, “18”: 2, “19”: 3,  “21”: 4, “23”: 1 |
| Pneumonia | Baseline | ICD J690 -  Pneumonitis due to inhalation of food and vomit | nominal | Diagnosis / ICD | 0% | “0”: 5743, “1”: 30, “2”: 2 |
|  |  | ICD J189 -  Pneumonia, unspecified organism | nominal | Diagnosis / ICD | 0% | “0”: 5718, “1”: 51, “2”: 5, “3”: 1 |
|  |  | Antiseptics Mouth Throat | nominal | Medications /  Medi-Span | 0% | “0”: 5451, “1”: 153, “2”: 61, “3”: 29, “4”: 14, “5”: 9, “6”: 9, “7”: 7, “8”: 5, “9”: 7, “10”: 3, “11”: 3, “12”: 2,  “13”: 1, “14”: 2, “15”: 2, “16”: 5,  “17”: 1, “18”: 1, “19”: 2, “21”: 1,  “22”: 1, “25”: 1, “26”: 3, “27”: 2 |
|  |  | ICD J9691 -  Respiratory failure, unspecified with hypoxia | nominal | Diagnosis / ICD | 0% | “0”: 5738, “1”: 36, “2”: 1 |
|  |  | ICD G7281 - Critical illness myopathy | nominal | Diagnosis / ICD | 0% | “0”: 5767, “1”: 7, “2”: 1 |
|  | Granular | ICD J690 - Pneumonitis due to inhalation of food and vomit | nominal | Diagnosis / ICD | 0% | “0”: 5743, “1”: 30, “2”: 2 |
|  |  | ICD J189 - Pneumonia, unspecified organism | nominal | Diagnosis / ICD | 0% | “0”: 5718, “1”: 51, “2”: 5, “3”: 1 |
|  |  | Antiseptics Mouth Throat | nominal | Medications /  Medi-Span | 0% | “0”: 5451, “1”: 153, “2”: 61, “3”: 29, “4”: 14, “5”: 9, “6”: 9, “7”: 7, “8”: 5, “9”: 7, “10”: 3, “11”: 3, “12”: 2,  “13”: 1, “14”: 2, “15”: 2, “16”: 5,  “17”: 1, “18”: 1, “19”: 2, “21”: 1,  “22”: 1, “25”: 1, “26”: 3, “27”: 2 |
|  |  | ICD J9691 - Respiratory failure, unspecified with hypoxia | nominal | Diagnosis / ICD | 0% | “0”: 5738, “1”: 36, “2”: 1 |
|  |  | ICD J9600 - Acute respiratory failure, unspecified whether with hypoxia or hypercapnia | nominal | Diagnosis / ICD | 0% | “0”: 5700, “1”: 73, “2”: 2 |
|  |  | ICD J9601 - Acute respiratory failure with hypoxia | nominal | Diagnosis / ICD | 0% | “0”: 5675, “1”: 99, “2”: 1 |
|  |  | ICD J80 - Acute respiratory distress syndrome | nominal | Diagnosis / ICD | 0% | “0”: 5755, “1”: 19, “2”: 1 |
|  |  | ICD A419 - Sepsis, unspecified organism | nominal | Diagnosis / ICD | 0% | “0”: 5590, “1”: 175, “2”: 9, “3”: 1 |
|  |  | ICD J9690 - Respiratory failure, unspecified, unspecified whether with hypoxia or hypercapnia | nominal | Diagnosis / ICD | 0% | “0”: 5705, “1”: 67, “2”: 3 |
|  |  | Chlorine Antiseptics | nominal | Medications /  Medi-Span | 0% | “0”: 5638, “1”: 59, “2”: 37, “3”: 19, “4”: 11, “5”: 4, “6”: 4, “7”: 1, “8”: 1, “9”: 1 |
|  |  | ICD R579 - Shock, unspecified | nominal | Diagnosis / ICD | 0% | “0”: 5746, “1”: 28, “2”: 1 |
|  |  | ICD 99731 - Ventilator associated pneumonia | nominal | Diagnosis / ICD | 0% | “0”: 5771, “2”: 4 |
|  |  | ICD L89150 - Pressure ulcer of sacral region, unstageable | nominal | Diagnosis / ICD | 0% | “0”: 5771, “1”: 4 |
|  |  | ICD S0081XA - Abrasion of other part of head, initial encounter | nominal | Diagnosis / ICD | 0% | “0”: 5772, “1”: 3 |
|  |  | ICD E870 - Hyperosmolality and hypernatremia | nominal | Diagnosis / ICD | 0% | “0”: 5736, “1”: 38, “2”: 1 |
|  |  | LAB 20578-1 median - Vancomycin, Blood | continuous | Laboratory Test Results / LOINC | 98.7% | Range: (1.1, 55.3), Mean: 17.0,  SD: 7.8 |
|  |  | LAB 3094-0 max - Urea Nitrogen (BUN), Blood | continuous | Laboratory Test Results / LOINC | 43.1% | Range: (3, 113), Mean: 19.1,  SD: 15.3 |
|  |  | LAB 20578-1 min - Vancomycin, Blood | continuous | Laboratory Test Results / LOINC | 98.7% | Range: (1.1, 42.2), Mean: 14.6,  SD: 7.0 |
|  |  | ICD Z9911 - Dependence on respirator [ventilator] status | nominal | Diagnosis / ICD | 0% | “0”: 5655, “1”: 117, “2”: 2, “3”: 1 |
|  |  | ICD 486 - Pneumonia, organism unspecified | nominal | Diagnosis / ICD | 0% | “0”: 5754, “1”: 17, “2”: 4 |
|  |  | LAB 2951-2 max - Sodium, Blood | continuous | Laboratory Test Results / LOINC | 43.1% | Range: (126.0, 158.5), Mean: 139.1,  SD: 3.3 |
|  |  | ICD B9689 - Other specified bacterial agents as the cause of diseases classified elsewhere | nominal | Diagnosis / ICD | 0% | “0”: 5702, “1”: 72, “2”: 1 |
|  |  | ICD J939 - Pneumothorax, unspecified | nominal | Diagnosis / ICD | 0% | “0”: 5758, “1”: 16, “3”: 1 |
|  |  | Antidiarrheal Agents Misc | nominal | Medications /  Medi-Span | 0% | “0”: 5643, “1”: 20, “2”: 23, “3”: 19, “4”: 18, “5”: 10, “6”: 8, “7”: 5, “8”: 6, “9”: 6, “10”: 3, “11”: 2, “13”: 2,  “14”: 1, “15”: 1, “16”: 2, “18”: 3,  “20”: 1, “25”: 1, “26”: 1 |
|  |  | LAB 2744-1 max - pH, Blood | continuous | Laboratory Test Results / LOINC | 87.0% | Range: (7.0, 7.6), Mean: 7.4,  SD: 0.07 |
|  | Grouped | Aspiration pneumonitis food/vomitus | nominal | Diagnosis / CCS | 0% | “0”: 5729, “1”: 43, “2”: 3 |
|  |  | Pneumonia except that caused by tuberculosis or sexually transmitted disease | nominal | Diagnosis / CCS | 0% | “0”: 5676, “1”: 89, “2”: 8, “3”: 1,  “5”: 1 |
|  |  | Mouth Throat Dental Agents | nominal | Medications /  Medi-Span | 0% | “0”: 5242, “1”: 281, “2”: 106, “3”: 39, “4”: 22, “5”: 13, “6”: 9, “7”: 12, “8”: 5, “9”: 7, “10”: 5, “11”: 6, “12”: 2,  “13”: 2, “14”: 2, “15”: 4, “16”: 5,  “18”: 1, “19”: 2, “21”: 1, “22”: 1,  “24”: 2, “25”: 1, “26”: 3, “27”: 2 |
|  |  | Shock | nominal | Diagnosis / CCS | 0% | “0”: 5602, “1”: 166, “2”: 7 |
|  |  | Septicemia except in labor | nominal | Diagnosis / CCS | 0% | “0”: 5506, “1”: 248, “2”: 17, “3”: 3, “5”: 1 |
|  |  | Respiratory failure insufficiency arrest adult | nominal | Diagnosis / CCS | 0% | “0”: 5248, “1”: 486, “2”: 36, “3”: 4, “4”: 1 |
|  |  | LAB LG47183-5 median - Vancomycin in Serum or Plasma | continuous | Laboratory Test Results / LOINC | 98.7% | Range: (1.1, 55.3), Mean: 17.0, SD: 7.8 |
|  |  | LAB LG1314-6 max - Urea nitrogen | continuous | Laboratory Test Results / LOINC | 43.1% | Range: (3, 113), Mean: 19.1, SD: 15.3 |
|  |  | LAB 19212-0 median - Carbon Dioxide (CO2), Blood | continuous | Laboratory Test Results / LOINC | 99.4% | Range: (30.0, 66.7), Mean: 43.0,  SD: 9.0 |
|  |  | LAB LG47183-5 min - Vancomycin in Serum or Plasma | continuous | Laboratory Test Results / LOINC | 98.7% | Range: (1.1, 42.2), Mean: 14.6, SD: 7.0 |
| Sepsis | Baseline | ICD A419 - Sepsis, unspecified organism | nominal | Diagnosis / ICD | 0% | “0”: 5590, “1”: 175, “2”: 9, “3”: 1 |
|  |  | ICD 0389 - Unspecified septicemia | nominal | Diagnosis / ICD | 0% | “0”: 5700, “1”: 70, “2”: 5 |
|  |  | Antiseptics Mouth Throat | nominal | Medications /  Medi-Span | 0% | “0”: 5451, “1”: 153, “2”: 61, “3”: 29, “4”: 14, “5”: 9, “6”: 9, “7”: 7, “8”: 5, “9”: 7, “10”: 3, “11”: 3, “12”: 2,  “13”: 1, “14”: 2, “15”: 2, “16”: 5,  “17”: 1, “18”: 1, “19”: 2, “21”: 1,  “22”: 1, “25”: 1, “26”: 3, “27”: 2 |
|  |  | ICD 78552 - Septic shock | nominal | Diagnosis / ICD | 0% | “0”: 5742, “1”: 32, “2”: 1 |
|  |  | ICD 99592 - Severe sepsis | nominal | Diagnosis / ICD | 0% | “0”: 5730, “1”: 43, “2”: 2 |
|  |  | LAB 2862-1 min - Albumin, Blood | continuous | Laboratory Test Results / LOINC | 67.9% | Range: (1.1, 6.0), Mean: 3.6, SD: 0.8 |
|  |  | ICD R6521 - Severe sepsis with septic shock | nominal | Diagnosis / ICD | 0% | “0”: 5700, “1”: 71, “2”: 4 |
|  |  | LAB 2862-1 median - Albumin, Blood | continuous | Laboratory Test Results / LOINC | 67.9% | Range: (1.3, 6.0), Mean: 3.7, SD: 0.7 |
|  |  | LAB 2862-1 mean - Albumin, Blood | continuous | Laboratory Test Results / LOINC | 67.9% | Range: (1.3, 6.0), Mean: 3.7, SD: 0.7 |
|  |  | LAB 3094-0 max - Urea Nitrogen (BUN), Blood | continuous | Laboratory Test Results / LOINC | 43.1% | Range: (3, 113), Mean: 19.1, SD: 15.3 |
|  |  | LAB 6690-2 max - White blood cell (WBC) count, Blood | continuous | Laboratory Test Results / LOINC | 44.3% | Range: (1.3, 47.7), Mean: 12.5,  SD: 5.8 |
|  |  | LAB 26464-8 max - White blood cell (WBC) count, Blood | continuous | Laboratory Test Results / LOINC | 44.2% | Range: (1.3, 47.7), Mean: 12.5,  SD: 5.8 |
|  |  | ICD K651 - Peritoneal abscess | nominal | Diagnosis / ICD | 0% | “0”: 5,674, “1”: 89, “2”: 10, “3”: 1, “4”: 1 |
|  |  | Low Molecular Weight Heparins | nominal | Medications /  Medi-Span | 0% | “0”: 3761, “1”: 258, “2”: 565, “3”: 351, “4”: 223, “5”: 156, “6”: 116, “7”: 78, “8”: 59, “9”: 27, “10”: 33, “11”: 29, “12”: 14, “13”: 21, “14”: 17, “15”: 9, “16”: 6, “17”: 8, “18”: 6, “19”: 10, “20”: 2, “21”: 6, “22”: 4, “23”: 5,  “24”: 3, “25”: 4, “26”: 2, “28”: 1,  “29”: 1 |
|  |  | LAB 26508-2 max - Band form neutrophils per 100 white blood cells, Blood | continuous | Laboratory Test Results / LOINC | 93.8% | Range: (0, 80), Mean: 20.9, SD: 17.9 |
|  |  | ICD 99591 - Sepsis | nominal | Diagnosis / ICD | 0% | “0”: 5708, “1”: 62, “2”: 5 |
|  |  | Nondepolarizing Muscle Relaxants | nominal | Medications /  Medi-Span | 0% | “0”: 1506, “1”: 4009, “2”: 194, “3”: 40, “4”: 16, “5”: 5, “6”: 3, “7”: 2 |
|  |  | Sodium | nominal | Medications /  Medi-Span | 0% | “0”: 4937, “1”: 484, “2”: 87, “3”: 39, “4”: 43, “5”: 33, “6”: 32, “7”: 15,  “8”: 24, “9”: 9, “10”: 9, “11”: 7,  “12”: 15, “13”: 10, “14”: 6, “15”: 2, “16”: 4, “17”: 4, “18”: 1, “19”: 4,  “20”: 1, “21”: 3, “23”: 2, “25”: 3,  “28”: 1 |
|  |  | Radiographic Contrast Media Iodinated | nominal | Medications /  Medi-Span | 0% | “0”: 4,364, “1”: 1,098, “2”: 214,  “3”: 63, “4”: 22, “5”: 6, “6”: 3, “7”: 5 |
|  |  | ICD R6520 - Severe sepsis without septic shock | nominal | Diagnosis / ICD | 0% | “0”: 5721, “1”: 51, “2”: 3 |
|  |  | Analgesics Other | nominal | Medications /  Medi-Span | 0% | “0”: 2310, “1”: 1124, “2”: 663,  “3”: 563, “4”: 301, “5”: 210, “6”: 143, “7”: 91, “8”: 73, “9”: 43, “10”: 42, “11”: 30, “12”: 38, “13”: 22, “14”: 19, “15”: 18, “16”: 18, “17”: 9, “18”: 8, “19”: 6, “20”: 2, “21”: 7, “22”: 2,  “23”: 5, “24”: 4, “25”: 3, “26”: 3,  “27”: 3, “29”: 1, “30”: 8, “31”: 6 |
|  |  | LAB 19218-7 min - Oxygen content, Blood | continuous | Laboratory Test Results / LOINC | 87.0% | Range: (7.1, 23.9), Mean: 14.4,  SD: 3.4 |
|  |  | LAB 20482-6 max - Granulocytes, Blood | continuous | Laboratory Test Results / LOINC | 57.2% | Range: (0.2, 40.6), Mean: 9.4,  SD: 5.3 |
|  |  | ICD Z9911 - Dependence on respirator [ventilator] status | nominal | Diagnosis / ICD | 0% | “0”: 5655, “1”: 117, “2”: 2, “3”: 1 |
|  |  | Proton Pump Inhibitors | nominal | Medications /  Medi-Span | 0% | “0”: 4153, “1”: 243, “2”: 254, “3”: 332, “4”: 179, “5”: 131, “6”: 102, “7”: 68, “8”: 70, “9”: 44, “10”: 27, “11”: 23, “12”: 28, “13”: 17, “14”: 7, “15”: 6, “16”: 11, “17”: 8, “18”: 9, “19”: 11, “20”: 12, “21”: 5, “22”: 5, “23”: 3, “24”: 3, “25”: 3, “26”: 4, “27”: 4,  “28”: 3, “29”: 5, “30”: 3, “31”: 2 |
|  | Granular | ICD A419 - Sepsis, unspecified organism | nominal | Diagnosis / ICD | 0% | “0”: 5590, “1”: 175, “2”: 9, “3”: 1 |
|  |  | ICD 0389 - Unspecified septicemia | nominal | Diagnosis / ICD | 0% | “0”: 5700, “1”: 70, “2”: 5 |
|  |  | Antiseptics Mouth Throat | nominal | Medications /  Medi-Span | 0% | “0”: 5451, “1”: 153, “2”: 61, “3”: 29, “4”: 14, “5”: 9, “6”: 9, “7”: 7, “8”: 5, “9”: 7, “10”: 3, “11”: 3, “12”: 2,  “13”: 1, “14”: 2, “15”: 2, “16”: 5,  “17”: 1, “18”: 1, “19”: 2, “21”: 1,  “22”: 1, “25”: 1, “26”: 3, “27”: 2 |
|  |  | ICD 78552 - Septic shock | nominal | Diagnosis / ICD | 0% | “0”: 5742, “1”: 32, “2”: 1 |
|  |  | ICD 99592 - Severe sepsis | nominal | Diagnosis / ICD | 0% | “0”: 5730, “1”: 43, “2”: 2 |
|  |  | LAB 2862-1 min - Albumin, Blood | continuous | Laboratory Test Results / LOINC | 67.9% | Range: (1.1, 6.0), Mean: 3.6,  SD: 0.8 |
|  |  | ICD R6521 - Severe sepsis with septic shock | nominal | Diagnosis / ICD | 0% | “0”: 5700, “1”: 71, “2”: 4 |
|  |  | LAB 2862-1 median - Albumin, Blood | continuous | Laboratory Test Results / LOINC | 67.9% | Range: (1.3, 6.0), Mean: 3.7,  SD: 0.7 |
|  |  | LAB 2862-1 mean - Albumin, Blood | continuous | Laboratory Test Results / LOINC | 67.9% | Range: (1.3, 6.0), Mean: 3.7,  SD: 0.7 |
|  |  | LAB 3094-0 max - Urea Nitrogen (BUN), Blood | continuous | Laboratory Test Results / LOINC | 43.1% | Range: (3, 113), Mean: 19.1,  SD: 15.3 |
|  |  | LAB 6690-2 max - White blood cell (WBC) count, Blood | continuous | Laboratory Test Results / LOINC | 44.3% | Range: (1.3, 47.7), Mean: 12.5,  SD: 5.8 |
|  |  | LAB 26464-8 max - White blood cell (WBC) count, Blood | continuous | Laboratory Test Results / LOINC | 44.2% | Range: (1.3, 47.7), Mean: 12.5,  SD: 5.8 |
|  |  | ICD K651 - Peritoneal abscess | nominal | Diagnosis / ICD | 0% | “0”: 5,674, “1”: 89, “2”: 10, “3”: 1, “4”: 1 |
|  |  | Low Molecular Weight Heparins | nominal | Medications /  Medi-Span | 0% | “0”: 3761, “1”: 258, “2”: 565, “3”: 351, “4”: 223, “5”: 156, “6”: 116, “7”: 78, “8”: 59, “9”: 27, “10”: 33, “11”: 29, “12”: 14, “13”: 21, “14”: 17, “15”: 9, “16”: 6, “17”: 8, “18”: 6, “19”: 10, “20”: 2, “21”: 6, “22”: 4, “23”: 5,  “24”: 3, “25”: 4, “26”: 2, “28”: 1,  “29”: 1 |
|  |  | ICD 99591 – Sepsis | nominal | Diagnosis / ICD | 0% | “0”: 5708, “1”: 62, “2”: 5 |
|  |  | Nondepolarizing Muscle Relaxants | nominal | Medications /  Medi-Span | 0% | “0”: 1506, “1”: 4009, “2”: 194, “3”: 40, “4”: 16, “5”: 5, “6”: 3, “7”: 2 |
|  |  | Sodium | nominal | Medications /  Medi-Span | 0% | “0”: 4937, “1”: 484, “2”: 87, “3”: 39, “4”: 43, “5”: 33, “6”: 32, “7”: 15,  “8”: 24, “9”: 9, “10”: 9, “11”: 7,  “12”: 15, “13”: 10, “14”: 6, “15”: 2, “16”: 4, “17”: 4, “18”: 1, “19”: 4,  “20”: 1, “21”: 3, “23”: 2, “25”: 3,  “28”: 1 |
|  |  | ICD R6520 - Severe sepsis without septic shock | nominal | Diagnosis / ICD | 0% | “0”: 5721, “1”: 51, “2”: 3 |
|  |  | Analgesics Other | nominal | Medications /  Medi-Span | 0% | “0”: 2310, “1”: 1124, “2”: 663,  “3”: 563, “4”: 301, “5”: 210, “6”: 143, “7”: 91, “8”: 73, “9”: 43, “10”: 42, “11”: 30, “12”: 38, “13”: 22, “14”: 19, “15”: 18, “16”: 18, “17”: 9, “18”: 8, “19”: 6, “20”: 2, “21”: 7, “22”: 2,  “23”: 5, “24”: 4, “25”: 3, “26”: 3,  “27”: 3, “29”: 1, “30”: 8, “31”: 6 |
|  |  | LAB 20482-6 max - Granulocytes, Blood | continuous | Laboratory Test Results / LOINC | 57.2% | Range: (0.2, 40.6), Mean: 9.4, SD: 5.3 |
|  |  | ICD Z9911 - Dependence on respirator [ventilator] status | nominal | Diagnosis / ICD | 0% | “0”: 5655, “1”: 117, “2”: 2, “3”: 1 |
|  |  | Proton Pump Inhibitors | nominal | Medications /  Medi-Span | 0% | “0”: 4153, “1”: 243, “2”: 254, “3”: 332, “4”: 179, “5”: 131, “6”: 102, “7”: 68, “8”: 70, “9”: 44, “10”: 27, “11”: 23, “12”: 28, “13”: 17, “14”: 7, “15”: 6, “16”: 11, “17”: 8, “18”: 9, “19”: 11, “20”: 12, “21”: 5, “22”: 5, “23”: 3, “24”: 3, “25”: 3, “26”: 4, “27”: 4,  “28”: 3, “29”: 5, “30”: 3, “31”: 2 |
|  |  | LAB 2518-9 max - Lactate, Blood | continuous | Laboratory Test Results / LOINC | 87.0% | Range: (0.3, 15.0), Mean: 2.1, SD: 2.1 |
|  |  | Opioid Agonists | nominal | Medications /  Medi-Span | 0% | “0”: 887, “1”: 2108, “2”: 854, “3”: 655, “4”: 347, “5”: 260, “6”: 150, “7”: 111, “8”: 77, “9”: 57, “10”: 44, “11”: 38, “12”: 31, “13”: 29, “14”: 15, “15”: 16, “16”: 12, “17”: 8, “18”: 10, “19”: 5, “20”: 12, “21”: 5, “22”: 6, “23”: 6,  “24”: 2, “25”: 5, “26”: 4, “27”: 3,  “28”: 4, “29”: 4, “30”: 3, “31”: 7 |
|  |  | ICD R000 - Tachycardia, unspecified | nominal | Diagnosis / ICD | 0% | “0”: 5450, “1”: 279, “2”: 32, “3”: 10, “4”: 1, “5”: 2, “6”: 1 |
|  | Grouped | Septicemia except in labor | nominal | Diagnosis / CCS | 0% | “0”: 5506, “1”: 248, “2”: 17, “3”: 3, “5”: 1 |
|  |  | Shock | nominal | Diagnosis / CCS | 0% | “0”: 5602, “1”: 166, “2”: 7 |
|  |  | Peritonitis and intestinal abscess | nominal | Diagnosis / CCS | 0% | “0”: 5488, “1”: 262, “2”: 23, “3”: 1, “5”: 1 |
|  |  | Mouth Throat Dental Agents | nominal | Medications /  Medi-Span | 0% | “0”: 5242, “1”: 281, “2”: 106, “3”: 39, “4”: 22, “5”: 13, “6”: 9, “7”: 12, “8”: 5, “9”: 7, “10”: 5, “11”: 6, “12”: 2,  “13”: 2, “14”: 2, “15”: 4, “16”: 5,  “18”: 1, “19”: 2, “21”: 1, “22”: 1,  “24”: 2, “25”: 1, “26”: 3, “27”: 2 |
|  |  | Minerals Electrolytes | nominal | Medications /  Medi-Span | 0% | “0”: 4311, “1”: 755, “2”: 196, “3”: 116, “4”: 70, “5”: 59, “6”: 58, “7”: 41,  “8”: 31, “9”: 19, “10”: 14, “11”: 9, “12”: 15, “13”: 13, “14”: 10, “15”: 11, “16”: 8, “17”: 4, “18”: 3, “19”: 6,  “20”: 5, “21”: 4, “22”: 2, “23”: 1,  “24”: 1, “25”: 4, “26”: 3, “27”: 3,  “28”: 2, “30”: 1 |
|  |  | Anticoagulants | nominal | Medications /  Medi-Span | 0% | “0”: 2777, “1”: 422, “2”: 492, “3”: 614, “4”: 297, “5”: 243, “6”: 199, “7”: 158, “8”: 105, “9”: 75, “10”: 66, “11”: 43, “12”: 40, “13”: 31, “14”: 35, “15”: 24, “16”: 18, “17”: 17, “18”: 16, “19”: 16, “20”: 14, “21”: 15, “22”: 2, “23”: 8,  “24”: 8, “25”: 9, “26”: 4, “27”: 7,  “28”: 8, “29”: 7, “30”: 4, “31”: 1 |
|  |  | LAB LG5465-2 min - Albumin | continuous | Laboratory Test Results / LOINC | 67.7% | Range: (1.1, 6.0), Mean: 3.6, SD: 0.8 |
|  |  | LAB LG32846-4 max - Granulocytes | continuous | Laboratory Test Results / LOINC | 56.3% | Range: (0.2, 43.9), Mean: 9.7, SD: 5.7 |
|  |  | LAB LG5465-2 median - Albumin | continuous | Laboratory Test Results / LOINC | 67.7% | Range: (1.3, 6.0), Mean: 3.7, SD: 0.7 |
|  |  | Ulcer Drugs | nominal | Medications /  Medi-Span | 0% | “0”: 2319, “1”: 1697, “2”: 310,  “3”: 430, “4”: 234, “5”: 146, “6”: 115, “7”: 106, “8”: 71, “9”: 66, “10”: 39, “11”: 29, “12”: 36, “13”: 23, “14”: 17, “15”: 16, “16”: 12, “17”: 12, “18”: 10, “19”: 17, “20”: 13, “21”: 5, “22”: 9, “23”: 1, “24”: 5, “25”: 6, “26”: 6,  “27”: 4, “28”: 5, “29”: 7, “30”: 6,  “31”: 3 |
| UTI | Baseline | ICD 99664 - Infection and inflammatory reaction due to indwelling urinary catheter | nominal | Diagnosis / ICD | 0% | “0”: 5773, “1”: 2 |
|  |  | LAB 5799-2 max - Leukocyte esterase, Urine | continuous | Laboratory Test Results / LOINC | 92.1% | Range: (0, 1), Mean: 0.2, SD: 0.4 |
|  |  | ICD 5990 - Urinary tract infection, site not specified | nominal | Diagnosis / ICD | 0% | “0”: 5706, “1”: 60, “2”: 6, “3”: 3 |
|  |  | LAB 5799-2 mean - Leukocyte esterase, Urine | continuous | Laboratory Test Results / LOINC | 92.1% | Range: (0, 1), Mean: 0.2, SD: 0.4 |
|  |  | ICD N390 - Urinary tract infection, site not specified | nominal | Diagnosis / ICD | 0% | “0”: 5616, “1”: 143, “2”: 13, “3”: 2, “4”: 1 |
|  |  | LAB 5821-4 mean - White blood cell (WBC) count, Urine sediment | continuous | Laboratory Test Results / LOINC | 85.0% | Range: (0, 217.3), Mean: 17.2,  SD: 39.9 |
|  |  | LAB 5821-4 median - White blood cell (WBC) count, Urine sediment | continuous | Laboratory Test Results / LOINC | 85.0% | Range: (0, 182), Mean: 16.8, SD: 39.9 |
|  |  | LAB 5821-4 min - White blood cell (WBC) count, Urine sediment | continuous | Laboratory Test Results / LOINC | 85.0% | Range: (0, 182), Mean: 14.0, SD: 37.3 |
|  |  | LAB 5799-2 median - Leukocyte esterase, Urine | continuous | Laboratory Test Results / LOINC | 92.1% | Range: (0, 1), Mean: 0.2, SD: 0.4 |
|  |  | ICD V463 - Wheelchair dependence | nominal | Diagnosis / ICD | 0% | “0”: 5767, “1”: 7, “3”: 1 |
|  |  | LAB 5821-4 max - White blood cell (WBC) count, Urine sediment | continuous | Laboratory Test Results / LOINC | 85.0% | Range: (0, 182), Mean: 20.6, SD: 45.8 |
|  |  | Urinary Analgesics | nominal | Medications /  Medi-Span | 0% | “0”: 5765, “2”: 6, “3”: 2, “4”: 1, “9”: 1 |
|  |  | LAB 5799-2 min - Leukocyte esterase, Urine | continuous | Laboratory Test Results / LOINC | 92.1% | Range: (0, 1), Mean: 0.2, SD: 0.4 |
|  |  | ICD 1840 - Malignant neoplasm of vagina | nominal | Diagnosis / ICD | 0% | “0”: 5774, “1”: 1 |
|  |  | ICD F59 - Unspecified behavioral syndromes associated with physiological disturbances and physical factors | nominal | Diagnosis / ICD | 0% | “0”: 5774, “1”: 1 |
|  |  | ICD I721 - Aneurysm of artery of upper extremity | nominal | Diagnosis / ICD | 0% | “0”: 5771, “1”: 3, “2”: 1 |
|  |  | ICD I7589 - Atheroembolism of other site | nominal | Diagnosis / ICD | 0% | “0”: 5774, “1”: 1 |
|  |  | ICD M19131 - Post-traumatic osteoarthritis, right wrist | nominal | Diagnosis / ICD | 0% | “0”: 5774, “1”: 1 |
|  |  | ICD M25331 - Other instability, right wrist | nominal | Diagnosis / ICD | 0% | “0”: 5774, “1”: 1 |
|  |  | ICD M79A11 - Nontraumatic compartment syndrome of right upper extremity | nominal | Diagnosis / ICD | 0% | “0”: 5774, “1”: 1 |
|  |  | ICD N10 - Acute pyelonephritis | nominal | Diagnosis / ICD | 0% | “0”: 5774, “1”: 1 |
|  |  | ICD S5010XA - Contusion of unspecified forearm, initial encounter | nominal | Diagnosis / ICD | 0% | “0”: 5774, “1”: 1 |
|  |  | ICD S55101A - Unspecified injury of radial artery at forearm level, right arm, initial encounter | nominal | Diagnosis / ICD | 0% | “0”: 5774, “1”: 1 |
|  |  | ICD S63591A - Other specified sprain of right wrist, initial encounter | nominal | Diagnosis / ICD | 0% | “0”: 5774, “1”: 1 |
|  |  | ICD V4362 - Elbow joint replacement | nominal | Diagnosis / ICD | 0% | “0”: 5774, “1”: 1 |
|  | Granular | ICD 99664 - Infection and inflammatory reaction due to indwelling urinary catheter | nominal | Diagnosis / ICD | 0% | “0”: 5773, “1”: 2 |
|  |  | ICD 5990 - Urinary tract infection, site not specified | nominal | Diagnosis / ICD | 0% | “0”: 5706, “1”: 60, “2”: 6, “3”: 3 |
|  |  | ICD N390 - Urinary tract infection, site not specified | nominal | Diagnosis / ICD | 0% | “0”: 5616, “1”: 143, “2”: 13, “3”: 2, “4”: 1 |
|  |  | LAB 5821-4 mean - White blood cell (WBC) count, Urine sediment | continuous | Laboratory Test Results / LOINC | 85.0% | Range: (0, 217.3), Mean: 17.2,  SD: 39.9 |
|  |  | LAB 5821-4 median - White blood cell (WBC) count, Urine sediment | continuous | Laboratory Test Results / LOINC | 85.0% | Range: (0, 182), Mean: 16.8, SD: 39.9 |
|  |  | LAB 5821-4 min - White blood cell (WBC) count, Urine sediment | continuous | Laboratory Test Results / LOINC | 85.0% | Range: (0, 182), Mean: 14.0, SD: 37.3 |
|  |  | ICD V463 - Wheelchair dependence | nominal | Diagnosis / ICD | 0% | “0”: 5767, “1”: 7, “3”: 1 |
|  |  | LAB 5821-4 max - White blood cell (WBC) count, Urine sediment | continuous | Laboratory Test Results / LOINC | 85.0% | Range: (0, 182), Mean: 20.6, SD: 45.8 |
|  |  | Urinary Analgesics | nominal | Medications /  Medi-Span | 0% | “0”: 5765, “2”: 6, “3”: 2, “4”: 1, “9”: 1 |
|  |  | ICD 1840 - Malignant neoplasm of vagina | nominal | Diagnosis / ICD | 0% | “0”: 5774, “1”: 1 |
|  |  | ICD 7813 - Lack of coordination | nominal | Diagnosis / ICD | 0% | “0”: 5772, “1”: 3 |
|  |  | ICD 92401 - Contusion of hip | nominal | Diagnosis / ICD | 0% | “0”: 5774, “1”: 1 |
|  |  | ICD 99527 - Other drug allergy | nominal | Diagnosis / ICD | 0% | “0”: 5774, “1”: 1 |
|  |  | ICD E9425 - Other vasodilators causing adverse effects in therapeutic use | nominal | Diagnosis / ICD | 0% | “0”: 5774, “1”: 1 |
|  |  | ICD F88 - Other disorders of psychological development | nominal | Diagnosis / ICD | 0% | “0”: 5774, “1”: 1 |
|  |  | ICD T50905S - Adverse effect of unspecified drugs, medicaments and biological substances, sequela | nominal | Diagnosis / ICD | 0% | “0”: 5774, “1”: 1 |
|  |  | ICD T8351XA - Infection and inflammatory reaction due to indwelling urinary catheter, initial encounter | nominal | Diagnosis / ICD | 0% | “0”: 5773, “1”: 2 |
|  |  | ICD V4459 - Other cystostomy | nominal | Diagnosis / ICD | 0% | “0”: 5774, “1”: 1 |
|  |  | Dibenzodiazepines | nominal | Medications /  Medi-Span | 0% | “0”: 5773, “8”: 1, “24”: 1 |
|  |  | ICD R300 - Dysuria | nominal | Diagnosis / ICD | 0% | “0”: 5724, “1”: 48, “2”: 3 |
|  |  | ICD 34400 - Quadriplegia, unspecified | nominal | Diagnosis / ICD | 0% | “0”: 5773, “1”: 1, “3”: 1 |
|  |  | Selective Estrogen Receptor Modulators SERMs | nominal | Medications /  Medi-Span | 0% | “0”: 5771, “1”: 1, “2”: 1, “4”: 1, “9”: 1 |
|  |  | ICD 59654 - Neurogenic bladder NOS | nominal | Diagnosis / ICD | 0% | “0”: 5763, “1”: 10, “2”: 2 |
|  |  | ICD 7292 - Neuralgia, neuritis, and radiculitis, unspecified | nominal | Diagnosis / ICD | 0% | “0”: 5767, “1”: 8 |
|  |  | ICD L0291 - Cutaneous abscess, unspecified | nominal | Diagnosis / ICD | 0% | “0”: 5764, “1”: 11 |
|  | Grouped | Urinary tract infections | nominal | Diagnosis / CCS | 0% | “0”: 5530, “1”: 215, “2”: 26, “3”: 2, “5”: 1, “6”: 1 |
|  |  | LAB LG40867-0 max - Leukocytes | continuous | Laboratory Test Results / LOINC | 79.8% | Range: (0, 182), Mean: 14.5, SD: 39.1 |
|  |  | LAB LG40867-0 mean – Leukocytes | continuous | Laboratory Test Results / LOINC | 79.8% | Range: (0, 217.3), Mean: 11.8,  SD: 33.2 |
|  |  | LAB LG40867-0 median – Leukocytes | continuous | Laboratory Test Results / LOINC | 79.8% | Range: (0, 182), Mean: 11.5, SD: 33.1 |
|  |  | LAB LG40868-8 max - Erythrocytes | continuous | Laboratory Test Results / LOINC | 79.5% | Range: (0, 1), Mean: 0.5, SD: 0.5 |
|  |  | Genitourinary symptoms and ill-defined conditions | nominal | Diagnosis / CCS | 0% | “0”: 5065, “1”: 607, “2”: 76, “3”: 20, “4”: 5, “6”: 1, “8”: 1 |
|  |  | LAB LG40868-8 mean – Erythrocytes | continuous | Laboratory Test Results / LOINC | 79.5% | Range: (0, 1), Mean: 0.4, SD: 0.5 |
|  |  | LAB LG40868-8 median – Erythrocytes | continuous | Laboratory Test Results / LOINC | 79.5% | Range: (0, 1), Mean: 0.4, SD: 0.5 |
|  |  | LAB LG40867-0 min – Leukocytes | continuous | Laboratory Test Results / LOINC | 79.8% | Range: (0, 182), Mean: 9.1, SD: 30.4 |
|  |  | LAB LG40868-8 min – Erythrocytes | continuous | Laboratory Test Results / LOINC | 79.5% | Range: (0, 1), Mean: 0.4, SD: 0.5 |

Table S3. Model calibration for top models selected for each outcome and dataset. The point estimates are based on 1000 bootstrapped iterations with 95% confidence intervals in parentheses.

| **Outcome** | **Metric** | **Baseline** | | **Granular** | | **Grouped** | |
| --- | --- | --- | --- | --- | --- | --- | --- |
|  |  | **Internal Validation** | **External Validation** | **Internal Validation** | **External Validation** | **Internal Validation** | **External Validation** |
| **SSI** | **Calibration-in-the-Large** | 4.64  (4.63, 4.65) | 3.11  (3.11, 3.12) | 2.49  (2.49, 2.50) | 0.95  (0.94, 0.95) | 3.87  (3.86, 3.89) | 3.75  (3.75, 3.75) |
|  | **Slope** | 0.56  (0.56, 0.57) | 0.82  (0.81, 0.82) | 0.62  (0.62, 0.62) | 0.63  (0.62, 0.63) | 0.63  (0.62, 0.63) | 0.49  (0.49, 0.49) |
|  | **Brier Score** | 0.10  (0.10, 0.10) | 0.07  (0.07, 0.07) | 0.09  (0.09, 0.09) | 0.07  (0.07, 0.07) | 0.09  (0.09, 0.09) | 0.09  (0.09, 0.09) |
| **Pneumonia** | **Calibration-in-the-Large** | 1.76  (1.75, 1.77) | 0.64  (0.64, 0.65) | 9.22  (9.20, 9.24) | 9.67  (9.67, 9.68) | 6.08  (6.00, 6.16) | 5.15  (5.12, 5.19) |
|  | **Slope** | 0.69  (0.67, 0.70) | 0.38  (0.37, 0.38) | 0.25  (0.24, 0.25) | 0.39  (0.39, 0.39) | 0.44  (0.43, 0.44) | 0.42  (0.42, 0.42) |
|  | **Brier Score** | 0.07  (0.07, 0.07) | 0.07  (0.07, 0.07) | 0.02  (0.02, 0.02) | 0.02  (0.02, 0.02) | 0.01  (0.01, 0.01) | 0.02  (0.02, 0.02) |
| **Sepsis** | **Calibration-in-the-Large** | 5.25  (5.24, 5.25) | 38.4  (38.4, 38.4) | 5.50  (5.49, 5.51) | 37.2  (37.0, 37.4) | 3.97  (3.96, 3.98) | 17.6  (17.6, 17.6) |
|  | **Slope** | 0.56  (0.56, 0.57) | 0.06  (0.06, 0.07) | 0.53  (0.53, 0.54) | 0.03  (0.03, 0.03) | 0.60  (0.59, 0.60) | 0.32  (0.32, 0.32) |
|  | **Brier Score** | 0.05  (0.05, 0.05) | 0.97  (0.97, 0.97) | 0.06  (0.06, 0.06) | 0.97  (0.97, 0.97) | 0.08  (0.08, 0.08) | 0.28  (0.28, 0.28) |
| **UTI** | **Calibration-in-the-Large** | 6.53  (6.51, 6.56) | 3.17  (3.16, 3.18) | 24.3  (24.2, 24.3) | 48.6  (48.6, 48.7) | 6.24  (6.22, 6.27) | 7.13  (7.12, 7.14) |
|  | **Slope** | 0.07  (0.07, 0.07) | 0.00002  (-0.002, 0.002) | 0.13  (0.13, 0.14) | 0.22  (0.22, 0.23) | 0.22  (0.22, 0.22) | 0.16  (0.16, 0.17) |
|  | **Brier Score** | 0.05  (0.05, 0.05) | 0.03  (0.03, 0.03) | 0.07  (0.07, 0.07) | 0.17  (0.17, 0.17) | 0.13  (0.13, 0.13) | 0.12  (0.12, 0.12) |

Table S4. Results of the top performing model for each outcome and dataset. The point estimates are based on 1000 bootstrapped iterations with 95% confidence intervals in parentheses. Difference-in-Difference (DiD); Support Vector Machine (SVM); Logistic Regression (LR); Random Forest (RF). Significantly positive DiD; ^a^ *P* < .001, ^b^ *P* < .01.

| **Outcome** | **Top Baseline Algorithm** | **Top Granular Algorithm** | **Top Grouped Algorithm** | **Metric** | **Baseline Internal Validation** | **Baseline External Validation** | **Granular Internal Validation** | **Granular External Validation** | **Grouped Internal Validation** | **Grouped External Validation** | **Baseline vs Granular DiD** | **Baseline vs Grouped DiD** | **Granular vs Grouped DiD** |
| --- | --- | --- | --- | --- | --- | --- | --- | --- | --- | --- | --- | --- | --- |
| SSI | SVM | LR | LR | AUC | 0.906  (0.904, 0.908) | 0.763  (0.762, 0.764) | 0.914  (0.913, 0.915) | 0.805  (0.805, 0.806) | 0.904  (0.903, 0.906) | 0.833  (0.833, 0.834) | 0.035^a^  (0.033, 0.037) | 0.072^a^  (0.070, 0.074) | 0.037^a^  (0.035, 0.039) |
|  |  |  |  | F1 | 0.501  (0.499, 0.503) | 0.300  (0.299, 0.302) | 0.554  (0.551, 0.556) | 0.371  (0.369, 0.372) | 0.476  (0.474, 0.478) | 0.376  (0.375, 0.376) | 0.017^a^  (0.014, 0.020) | 0.100^a^  (0.097, 0.103) | 0.082^a^  (0.079, 0.085) |
|  |  |  |  | Precision | 0.364  (0.363, 0.366) | 0.475  (0.473, 0.476) | 0.429  (0.426, 0.432) | 0.504  (0.502, 0.506) | 0.337  (0.335, 0.338) | 0.270  (0.270, 0.271) | -0.036  (-0.039,  -0.033) | -0.177  (-0.180,  -0.175) | -0.143  (-0.146,  -0.140) |
|  |  |  |  | Sensitivity | 0.804  (0.802, 0.807) | 0.220  (0.219, 0.221) | 0.784  (0.780, 0.787) | 0.293  (0.292, 0.294) | 0.815  (0.811, 0.819) | 0.615  (0.614, 0.617) | 0.096^a^  (0.092, 0.100) | 0.386^a^  (0.382, 0.391) | 0.290^a^  (0.286, 0.294) |
|  |  |  |  | Specificity | 0.925  (0.925, 0.926) | 0.987  (0.987, 0.987) | 0.945  (0.944, 0.945) | 0.985  (0.985, 0.985) | 0.915  (0.914, 0.915) | 0.914  (0.914, 0.914) | -0.022  (-0.022,  -0.021) | -0.063  (-0.064,  -0.062) | -0.042  (-0.042,  -0.041) |
|  |  |  |  | Accuracy | 0.919  (0.919, 0.920) | 0.950  (0.949, 0.950) | 0.937  (0.936, 0.937) | 0.951  (0.951, 0.951) | 0.910  (0.909, 0.910) | 0.899  (0.899, 0.899) | -0.016  (-0.016,  -0.015) | -0.041  (-0.042,  -0.040) | -0.025  (-0.026,  -0.025) |
|  |  |  |  | Balanced Accuracy | 0.865  (0.864, 0.866) | 0.604  (0.603, 0.604) | 0.864  (0.862, 0.866) | 0.639  (0.639, 0.640) | 0.865  (0.863, 0.867) | 0.765  (0.764, 0.765) | 0.037^a^  (0.035, 0.039) | 0.162^a^  (0.160, 0.164) | 0.124^a^  (0.122, 0.126) |
| Pneumonia | LR | LR | SVM | AUC | 0.953  (0.949, 0.957) | 0.683  (0.682, 0.685) | 0.990  (0.990, 0.991) | 0.948  (0.947, 0.948) | 0.994  (0.994, 0.995) | 0.973  (0.973, 0.974) | 0.226^a^  (0.223, 0.229) | 0.250^a^  (0.247, 0.252) | 0.022^a^  (0.021, 0.023) |
|  |  |  |  | F1 | 0.504  (0.498, 0.509) | 0.302  (0.299, 0.305) | 0.379  (0.371, 0.386) | 0.359  (0.357, 0.360) | 0.456  (0.452, 0.461) | 0.467  (0.465, 0.468) | 0.186^a^  (0.179, 0.193) | 0.212^a^  (0.206, 0.218) | 0.031^a^  (0.026, 0.037) |
|  |  |  |  | Precision | 0.377  (0.372, 0.382) | 0.393  (0.389, 0.397) | 0.262  (0.256, 0.267) | 0.260  (0.259, 0.261) | 0.305  (0.301, 0.309) | 0.321  (0.320, 0.322) | -0.014  (-0.020,  -0.008) | 0.0001  (-0.005, 0.006) | 0.018^a^  (0.014, 0.023) |
|  |  |  |  | Sensitivity | 0.770  (0.763, 0.777) | 0.245  (0.243, 0.248) | 0.697  (0.684, 0.710) | 0.579  (0.576, 0.581) | 0.921  (0.916, 0.926) | 0.853  (0.851, 0.856) | 0.412^a^  (0.400, 0.423) | 0.456^a^  (0.447, 0.464) | 0.056^a^  (0.046, 0.065) |
|  |  |  |  | Specificity | 0.990  (0.990, 0.990) | 0.996  (0.996, 0.996) | 0.985  (0.985, 0.985) | 0.982  (0.981, 0.982) | 0.984  (0.983, 0.984) | 0.980  (0.979, 0.982) | -0.009  (-0.009,  -0.009) | -0.010  (-0.010,  -0.009) | -0.0008  (-0.001,  -0.0005) |
|  |  |  |  | Accuracy | 0.988  (0.988, 0.989) | 0.987  (0.987, 0.987) | 0.983  (0.982, 0.983) | 0.977  (0.977, 0.977) | 0.983  (09.83, 0.984) | 0.978  (0.978, 0.978) | -0.004  (-0.005,  -0.004) | -0.004  (-0.004,  -0.004) | 0.0006^a^  (0.0004, 0.0009) |
|  |  |  |  | Balanced Accuracy | 0.880  (0.876, 0.884) | 0.621  (0.619, 0.622) | 0.841  (0.834, 0.847) | 0.780  (0.779, 0.781) | 0.953  (0.950, 0.955) | 0.917  (0.916, 0.918) | 0.201^a^  (0.195, 0.207) | 0.223^a^  (0.219, 0.227) | 0.027^a^  (0.023, 0.032) |
| Sepsis | LR | LR | RF | AUC | 0.964  (0.963, 0.964) | 0.890  (0.889, 0.891) | 0.949  (0.947, 0.951) | 0.889  (0.889, 0.890) | 0.948  (0.946, 0.949) | 0.883  (0.883, 0.884) | 0.015^a^  (0.013, 0.017) | 0.008^a^  (0.007, 0.010) | -0.003  (-0.005,  -0.001) |
|  |  |  |  | F1 | 0.469  (0.467, 0.472) | 0.050  (0.050, 0.050) | 0.446  (0.442, 0.449) | 0.051  (0.051, 0.051) | 0.419  (0.416, 0.422) | 0.092  (0.092, 0.093) | 0.026^a^  (0.023, 0.028) | 0.091^a^  (0.089, 0.093) | 0.071^a^  (0.069, 0.073) |
|  |  |  |  | Precision | 0.325  (0.323, 0.327) | 0.026  (0.026, 0.026) | 0.305  (0.302, 0.308) | 0.026  (0.026, 0.026) | 0.273  (0.271, 0.274) | 0.049  (0.049, 0.049) | 0.021^a^  (0.019, 0.023) | 0.074^a^  (0.072, 0.076) | 0.057^a^  (0.055, 0.059) |
|  |  |  |  | Sensitivity | 0.849  (0.845, 0.853) | 0.990  (0.990, 0.991) | 0.831  (0.826, 0.836) | 1.000  (1.000, 1.000) | 0.906  (0.903, 0.910) | 0.910  (0.909, 0.911) | 0.032^a^  (0.027, 0.036) | -0.139  (-0.143,  -0.135) | -0.163  (-0.167,  -0.159) |
|  |  |  |  | Specificity | 0.944  (0.944, 0.944) | 0.006  (0.006, 0.006) | 0.940  (0.939, 0.940) | 0.000  (0.000,  0.000) | 0.923  (0.923, 0.924) | 0.527  (0.526, 0.527) | -0.002  (-0.002,  -0.001) | 0.541^a^  (0.541, 0.542) | 0.543^a^  (0.543, 0.544) |
|  |  |  |  | Accuracy | 0.941  (0.941, 0.941) | 0.031  (0.031, 0.031) | 0.937  (0.936, 0.937) | 0.026  (0.026, 0.026) | 0.923  (0.922, 0.923) | 0.537  (0.536, 0.537) | -0.0007  (-0.001,  -0.0001) | 0.523^a^  (0.523, 0.524) | 0.525^a^  (0.524, 0.525) |
|  |  |  |  | Balanced Accuracy | 0.897  (0.894, 0.899) | 0.498  (0.498, 0.498) | 0.885  (0.883, 0.888) | 0.500  (0.500, 0.500) | 0.915  (0.913, 0.917) | 0.718  (0.718, 0.719) | 0.015^a^  (0.013, 0.017) | 0.201^a^  (0.199, 0.203) | 0.190^a^  (0.188, 0.192) |
| UTI | SVM | RF | LR | AUC | 0.898  (0.895, 0.900) | 0.886  (0.885, 0.887) | 0.932  (0.929, 0.934) | 0.872  (0.871, 0.874) | 0.936  (0.934, 0.939) | 0.929  (0.928, 0.930) | -0.049  (-0.052,  -0.045) | 0.006^b^  (0.002, 0.009) | 0.051^a^  (0.048, 0.055) |
|  |  |  |  | F1 | 0.153  (0.148, 0.158) | 0.063  (0.061, 0.064) | 0.169  (0.167, 0.171) | 0.121  (0.120, 0.121) | 0.244  (0.241, 0.246) | 0.225  (0.224, 0.226) | 0.039^a^  (0.035, 0.043) | 0.073^a^  (0.068, 0.077) | 0.030^a^  (0.027, 0.032) |
|  |  |  |  | Precision | 0.100  (0.097, 0.103) | 0.045  (0.044, 0.046) | 0.095  (0.093, 0.096) | 0.065  (0.065, 0.065) | 0.142  (0.140, 0.144) | 0.133  (0.133, 0.134) | 0.024^a^  (0.021, 0.026) | 0.047^a^  (0.045, 0.050) | 0.021^a^  (0.020, 0.023) |
|  |  |  |  | Sensitivity | 0.331  (0.321, 0.340) | 0.103  (0.101, 0.106) | 0.798  (0.788, 0.809) | 0.840  (0.837, 0.842) | 0.868  (0.860, 0.876) | 0.711  (0.707, 0.713) | 0.261^a^  (0.251, 0.271) | 0.071^a^  (0.061, 0.081) | -0.200  (-0.209,  -0.191) |
|  |  |  |  | Specificity | 0.974  (0.974, 0.974) | 0.982  (0.982, 0.982) | 0.933  (0.933, 0.933) | 0.901  (0.901, 0.902) | 0.954  (0.953, 0.954) | 0.962  (0.962, 0.962) | -0.040  (-0.040,  -0.039) | 0.0003  (-0.0001, 0.0007) | 0.040^a^  (0.040, 0.041) |
|  |  |  |  | Accuracy | 0.968  (0.968, 0.969) | 0.975  (0.975, 0.975) | 0.932  (0.931, 0.932) | 0.901  (0.901, 0.901) | 0.953  (0.953, 0.953) | 0.960  (0.960, 0.960) | -0.038  (-0.038,  -0.037) | 0.0006^b^  (0.0001, 0.001) | 0.038^a^  (0.038, 0.039) |
|  |  |  |  | Balanced Accuracy | 0.652  (0.647, 0.657) | 0.543  (0.541, 0.544) | 0.866  (0.860, 0.871) | 0.870  (0.869, 0.872) | 0.911  (0.907, 0.915) | 0.836  (0.835, 0.838) | 0.111^a^  (0.105, 0.116) | 0.036^a^  (0.031, 0.041) | -0.080  (-0.085,  -0.076) |

### Figures

Figure S1. Calibration plot for baseline and grouped SSI models.


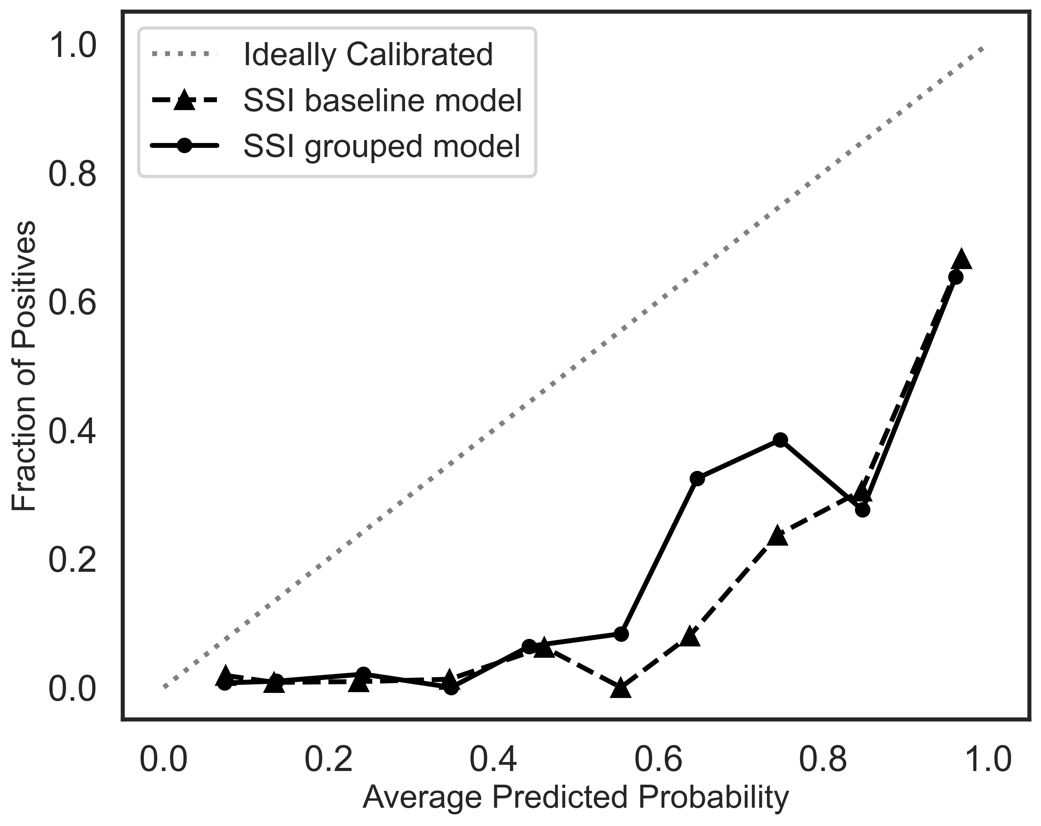


Figure S2. Calibration plot for baseline and grouped Pneumonia models.


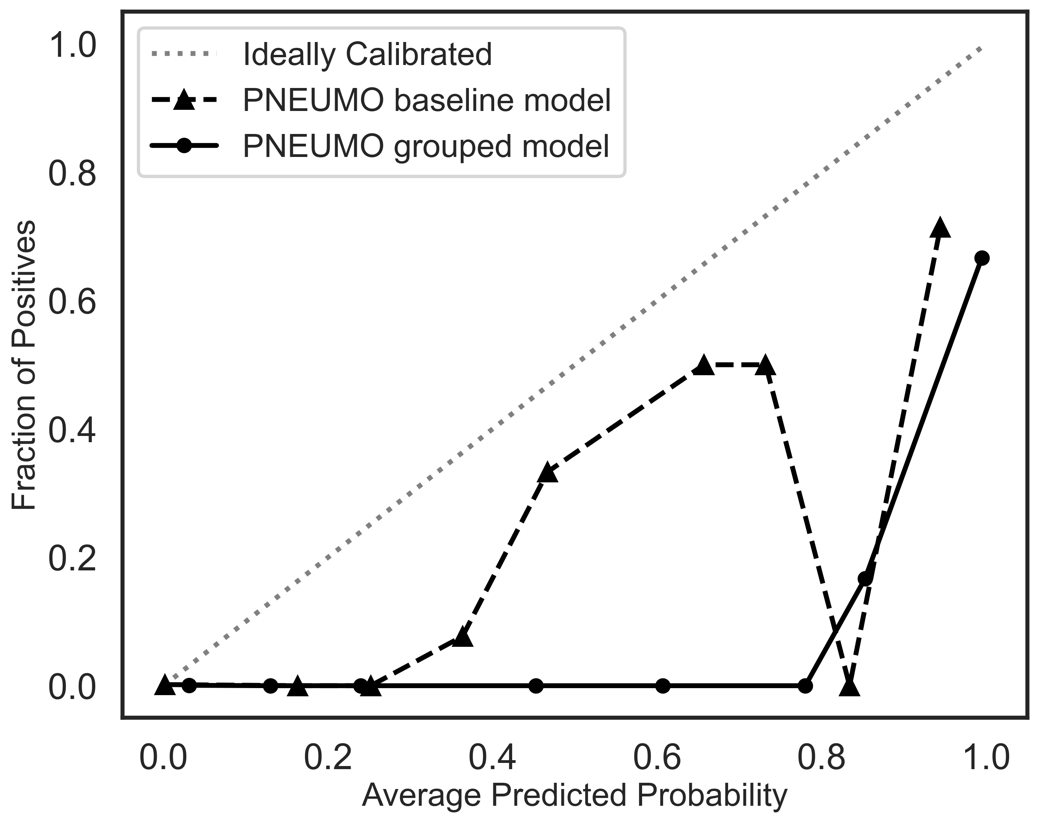


Figure S3. Calibration plot for baseline and grouped Sepsis models.


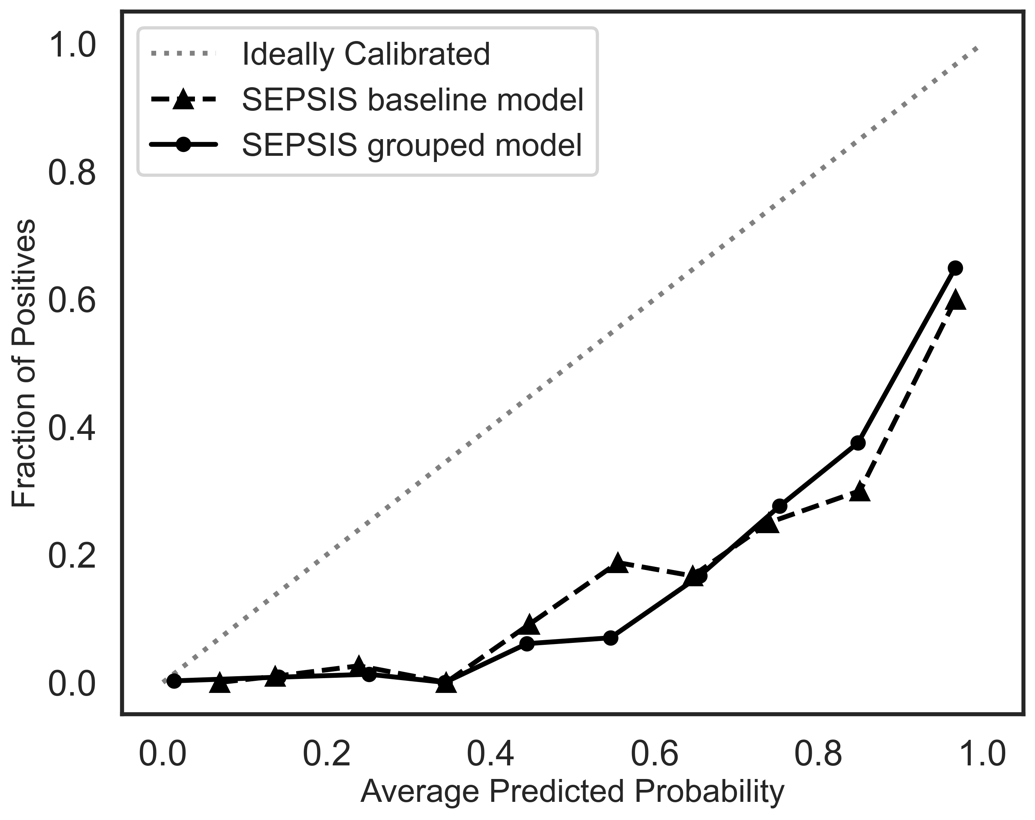


Figure S4. Calibration plot for baseline and grouped UTI models.


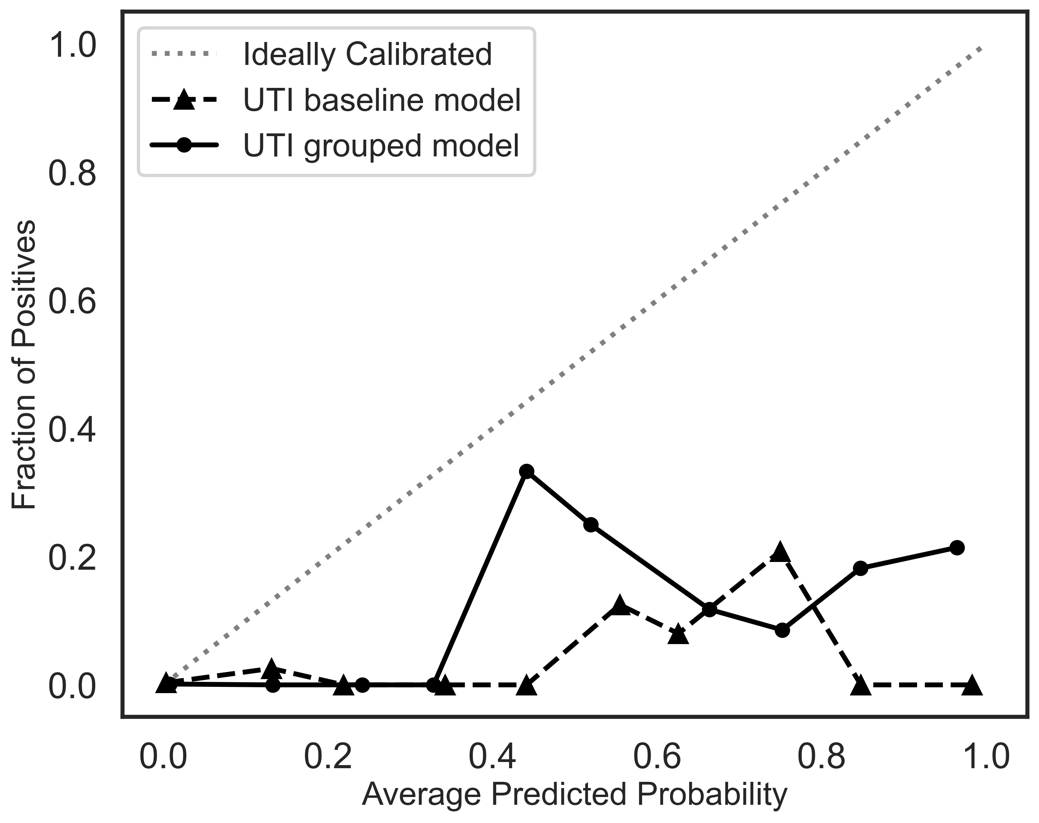


Figure S5. STARD flow diagram of patient data through the baseline model, predicting SSI.

Figure S6. STARD flow diagram of patient data through the granular model, predicting SSI.

Figure S7. STARD flow diagram of patient data through the grouped model, predicting SSI.

Figure S8. STARD flow diagram of patient data through the baseline model, predicting sepsis.

Figure S9. STARD flow diagram of patient data through the granular model, predicting sepsis.

Figure S10. STARD flow diagram of patient data through the grouped model, predicting sepsis.

Figure S11. STARD flow diagram of patient data through the baseline model, predicting pneumonia.

Figure S12. STARD flow diagram of patient data through the granular model, predicting pneumonia.

Figure S13. STARD flow diagram of patient data through the grouped model, predicting pneumonia.

Figure S14. STARD flow diagram of patient data through the baseline model, predicting UTI.

Figure S15. STARD flow diagram of patient data through the granular model, predicting UTI.

Figure S16. STARD flow diagram of patient data through the grouped model, predicting UTI.

Figure S17. Difference-in-difference (DiD) found in the area under the receiver operating characteristic curve (AUC) and F1-scores (F1) for all outcomes. A positive DiD indicated the grouped model resulted in less of a performance drop when compared to the baseline model.
